# Unexpected Genomic Variability in Clinical and Environmental Strains of the Pathogenic Yeast *Candida parapsilosis*

**DOI:** 10.1093/gbe/evt185

**Published:** 2013-11-20

**Authors:** Leszek P. Pryszcz, Tibor Németh, Attila Gácser, Toni Gabaldón

**Affiliations:** ^1^Bioinformatics and Genomics Programme, Centre for Genomic Regulation (CRG), Barcelona, Spain; ^2^Universitat Pompeu Fabra (UPF), Barcelona, Spain; ^3^Department of Microbiology, University of Szeged, Szeged, Hungary; ^4^Institució Catalana de Recerca I Estudis Avançats (ICREA), Barcelona, Spain

**Keywords:** genome comparison, recombination, pathogens, *Candida*

## Abstract

Invasive candidiasis is the most commonly reported invasive fungal infection worldwide. Although *Candida albicans* remains the main cause, the incidence of emerging *Candida* species, such as *C. parapsilosis* is increasing. It has been postulated that *C. parapsilosis* clinical isolates result from a recent global expansion of a virulent clone. However, the availability of a single genome for this species has so far prevented testing this hypothesis at genomic scales. We present here the sequence of three additional strains from clinical and environmental samples. Our analyses reveal unexpected patterns of genomic variation, shared among distant strains, that argue against the clonal expansion hypothesis. All strains carry independent expansions involving an arsenite transporter homolog, pointing to the existence of directional selection in the environment, and independent origins of the two clinical isolates. Furthermore, we report the first evidence for the existence of recombination in this species. Altogether, our results shed new light onto the dynamics of genome evolution in *C. parapsilosis*.

## Introduction

*Candida* spp. comprise several species that are part of the human commensal flora. Some *Candida* species can cause superficial or systemic infections, particularly under conditions of immune suppression. During the last three decades, invasive candidiasis has become the most commonly diagnosed severe yeast infection worldwide (Trofa et al. 2008; van Asbeck et al. 2009). Many *Candida* species are reported to cause nosocomial infections in hospitalized patients (Pfaller et al. 2010), especially affecting elderly patients receiving prolonged medical attendance (Jain et al. 2011), low birthweight neonates (Benjamin et al. 2003; Clerihew et al. 2007; Trofa et al. 2008), and patients with superficial injuries or with antimicrobial or steroid treatment (Trofa et al. 2008). In this regard, *Candida* spp. can be found on the hands of every third healthcare worker and are known as the fourth most commonly isolated microorganism from blood cultures of hospitalized patients (Jain et al. 2011). Although *Candida albicans* remains the most prevalent species in candidiasis, the relevance of other *Candida* spp*.*, such as *C. glabrata*, *C. parapsilosis*, and *C. tropicalis*, have significantly increased in recent years (Trofa et al. 2008; Pfaller et al. 2010). Of these, the epidemiology of *C. parapsilosis* shows remarkable differences compared with *C. albicans*. *Candida parapsilosis* causes a disproportionate amount of disease in neonates, which may in part be a reflection of its unique genetic background. Indeed, the incidence of *C. parapsilosis* in these patients can significantly outrank that of *C. albicans*, depending on the period and geographic area (Zaoutis 2010).

Understanding the molecular basis of pathogenesis in the different *Candida* spp., as well as the evolutionary emergence of their virulence requires the identification of shared and species- and strain-specific virulence factors, a task that has been eased by the rapid development of high-throughput molecular techniques, including DNA sequencing. Earlier work has highlighted the importance for *Candida* virulence of particular genes, such as secreted proteinases, lipases, phospholipases, and adhesins, as well as phenotypic properties, such as the ability to form biofilms and/or hyphae (Zakikhany et al. 2008; Miramon et al. 2013). Interestingly, studies performed during the last few years have revealed notable genetic differences not only between *Candida* species but also between different isolates of the same species (Butler et al. 2009). These intraspecies differences have been mostly investigated in *C. albicans*, where they have been linked to changes in drug susceptibility profiles (Morio et al. 2010), or to different degrees of virulence (Sampaio et al. 2010). Although the comparison of the genomes of different *C. albicans* strains have produced highly valuable data, the comparative genetics of different strains of emerging *Candida* species like *C. parapsilosis* has so far never been performed. To date, the sequence of a single strain of *C. parapsilosis* (CDC317) is available (Butler et al. 2009; Guida et al. 2011), limiting our understanding of the genomic diversity within this important pathogen. Based on the observation that *C. parapsilosis* isolates are extremely difficult to differentiate using standard techniques (Kuhn et al. 2004; Tavanti et al. 2010) and that the level of heterozygosis between chromosomes in the sequenced isolate was 25- to 70-fold lower than in other *Candida* species (Kuhn et al. 2004; Butler et al. 2009; Hensgens et al. 2009; Tavanti et al. 2010), it has been suggested that *C. parapsilosis* may have undergone a very recent population bottleneck (Sai et al. 2011). In addition, it was speculated that this recent clonal expansion of a strain with increased virulence—when compared with the closest relatives *C. orthopsilosis* and *C. metapsilosis*—may be related with the recent loss of a mating partner (MTLα) and the degeneration of the MTL**a** idiomorph (Logue et al. 2005; Sai et al. 2011). Despite these lines of evidence pointing to a recent global spread of a single *C. parapsilosis* clone, more evidence is needed from the analyses of a broader set of genomic markers. Here, we set out to investigate the actual variability among *C. parapsilosis* isolates by using a whole genome sequencing approach. For this, we sequenced three different strains including the clinical isolate GA1, which has been extensively used in the study of *C. parapsilosis* virulence (Gacser et al. 2005, 2007), and two environmental isolates from different geographic locations (CBS1954, CBS6318). Our results confirm the previously reported low levels of heterozygosity in this species but point to a high level of genomic variability, including copy number variations (CNVs), rearrangements, and point mutations. This level of genomic variability is unexpected considering the difficulty of distinguishing among strains using standard molecular techniques and underscores the resolution provided by massive sequencing techniques. Importantly, some genomic variations shared by distant strains challenge the idea that all clinical strains diverged vertically from a single clone. Altogether, our findings argue against the hypothesis of a clonal expansion of clinical isolates of this species and show, for the first time, evidence of genomic recombination in this pathogenic species.

## Materials and Methods

### DNA Extraction

Cultures were incubated in an orbital shaker (200 rpm) overnight at 30 °C in 2 ml Yeast Peptone Dextrose (YPD) medium (0.5% yeast extract, 1% peptone, and 1% glucose) supplemented with 1% penicillin–streptomycin (Sigma). Yeast cells were centrifuged at 900 × g for 5 min and were washed twice with 1 × sterile phosphate buffered saline (PBS) medium. The pellett was resuspended in 500 μl lysis buffer (1 w/v% SDS, 50 mM EDTA, and 100 mM TRIS pH = 8), and 500 μl of glass beads was added to the cells and shacked in a vortex for 3 min. Ammonium acetate was added (275 μl 7 M, 65 °C, 5 min), and the samples were cooled on ice for 5 min. Then, 500 μl of chloroform–isoamylalcohol (24:1) was added to the mixture and was centrifuged for 10 min at17,000 × g. The upper phase was transferred to a new tube, and the previous step was repeated. Then, the upper phase was mixed with 500 μl isopropanol in a new tube and placed at −20 °C for 5 min. The solution was centrifuged at 17,000 × g for 10 min and the supernatant discarded. The pellet was washed twice with 500 μl 70% ethanol, dried, and suspended in 100 μl bidistilled water containing RN-ase (Sigma).

### RNA Isolation

Cultures were grown as indicated above. For RNA isolation, Qiagen RNeasy Plant Mini Plus Kit was used. Yeasts were harvested by centrifugation (220 g, 5 min, 4 °C) and washed with 1× sterile PBS. Cells were suspended in 650 µl RNAeasy Lipid Tissue (RLT) buffer (supplemented with 1% β-mercaptoethanol) and were vortex mixed vigorously with 500 µl glass beads in 2 ml micocentrifuge tubes for 3 min. The supernatant was centrifuged at 16,000 × g for 1 min, transferred, and precipitated with 70% ethanol on ice. Every following steps were performed according to the manufacturer’s instructions. On-column DNase digestion was performed using Quiagen RNase-Free DNase Set based on the provided protocol.

### Genome Sequencing

The three strains were sequenced at the genomics facility at Centre for Genomic Regulation (CRG), using either Illumina Genome Analyzer IIx (GA) or Hiseq2000 (HS) sequencing platforms (see table 1). DNA was fragmented by nebulization or in Covaris to a size ∼400–600 bp. After shearing, the ends of DNA fragments were blunted with T4 DNA polymerase and Klenow fragment (New England Biolabs). Then, DNA was purified with a QIAquick PCR purification kit (Qiagen). Thereafter, 3′-adenylation was performed by incubation with dATP and 3′-5′-exo-Klenow fragment (New England Biolabs). DNA was purified using MinElute spin columns (Qiagen), and double-stranded Illumina paired-end adapters were ligated to the DNA using rapid T4 DNA ligase (New England Biolabs). After another purification step, adapter-ligated fragments were enriched, and adapters were extended by selective amplification in an 18-cycle polymerase chain reaction (PCR) using Phusion DNA polymerase (Finnzymes). Libraries were quantified and loaded into Illumina flow cells at concentrations of 7–20 pM (Genome Analyzer IIx; GA) or 1.4–1.75 pM (HiSeq 2000; HS). Cluster generation was performed in an Illumina cluster station (GA) or in a cBOT (HS). Sequence runs of 2 × 75 (GA) to 2 × 100 (HS) cycles were performed on the sequencing instruments. Base calling was performed using an Illumina pipeline software. In multiplexed libraries, we used 4 bp internal indices (5′ indexed sequences). Deconvolution was performed using the CASAVA software (Illumina).

**Table 1:** Basic Strain and Assembly Statistics for the Genomes Obtained within This Work and the Reference *C. parapsilosis* Strain

Strain	Origin	Library	Number of Reads (millions)	Coverage (*x*)	Scaffolds	Assembly Size (kb)	Heterozygous Sites (%)	Genes
CBS1954	Environmental, olive fruit, Italy	76 bp; GAIIx; paired at 300 bp	87.51	510	20	13,107	0.00637	5,696
CBS6318	Environmental, healthy skin, USA	76 bp; GAIIx; paired at 300 bp	42.45	248	24	13,138	0.00457	5,677
GA1	Clinical, human blood, Germany	96 bp; HiSeq; paired at 600 bp	36.51	269	27	13,017	0.00717	5,692
CDC317	Clinical, skin, USA	Sanger	0.23	12	8	13,030	0.01576	5,836

Note.—For each strain, the table provides, in this order: strain name; geographical origin and sampling context; sequencing strategy, indicating read length, sequencing platform and insert size (in case of paired reads); number of reads obtained, average coverage (*x* fold); number of scaffolds; total assembly size; fraction of heterozygous sites; number of predicted genes.

### Genome Assembly

Reads were preprocessed before assembly to trim at the first undetermined base or at the first base having a PHRED quality score below 10. The pairs with one (or both) read shorter than 31 bases after trimming were excluded from the assembly process. Paired-end reads were assembled into supercontigs using SOAPdenovo (Li et al. 2008) version 1.05. We tested multiple combinations of parameters, including Kmer ranging from 31 to 91 stepping by two, and mergeLevel from 0 to 3. The best final assembly was chosen based on multiple assembly quality measures including *N*50, number of gaps, and number of supercontigs longer than 1 kb. Gaps were filled using GapCloser included in the SOAPdenovo package. Finally, supercontigs were scaffolded on *C. parapsilosis* CDC317 chromosomes using Oslay (Richter et al. 2007) version 1.0.

### Genome Annotation

Genes were predicted with Augustus version 2.5.5 (Stanke et al. 2006) using *C. parapsilosis* CDC317 gene models for training (Guida et al. 2011). RNA-Seq reads (accession number ERP004376) were used as hints for detection of exon–intron boundaries. Finally, genes were annotated with: 1) putative functions derived from Gene Ontology (GO) terms transferred from one-to-one orthologs in model species, that is, *C. albicans* or *Saccharomyces cerevisiae*, based on predictions from the MetaPhORs (Pryszcz et al. 2011) webserver and 2) protein domains from PFAM (Bateman et al. 2004) predicted with HMMER (Eddy 2011) version 3.0.3.

### Detection of Single Nucleotide Polymorphisms and Genomic Recombination

Reads were aligned onto *C. parapsilosi*s CDC317 chromosomes using Bowtie2 (Langmead and Salzberg 2012), with “very sensitive local alignment” mode. Single nucleotide polymorphisms (SNPs) and insertions and deletions (INDELs) were called using GATK (McKenna et al. 2010) version 2.1–13. We filtered out clusters of three variants within 20 bases and low-quality variants, as described in GATK documentation (QD < 2.0 || MQ < 40 || FS > 60.0 || HaplotypeScore > 13.0 || MQRankSum < −12.5 || ReadPosRankSum < −8.0). Subsequently, we divided variants into two groups: homo- and heterozygous. All analyses shown here are based only on homozygous variants. Regions having at least three consecutive homozygous mutations per 200 bp were defined as mutational hotspots. Finally, we screened full-length chromosome alignments of the four *C. parapsilosis* strains to detect genomic recombination using RDP3 (Martin et al. 2010). RDP3 was also applied to find gene conversion between agglutinin-like sequence (ALS) family members.

### Detection of Structural Variants

Structural variants were first detected using the Delly package (Rausch et al. 2012). Delly exploits variation in pair-end/mate-pair reads distance and orientation to detect duplications, deletions, inversions, and translocations. In fact, Delly is unable to detect variants that are not captured by such libraries, that is, insertions larger than insert size. Of note, heuristics applied in short read aligners limit alignment of pairs within a given distance range. As a result, large deletions are often difficult to detect with this technique. Lastly, this method is prone to false positives. To circumvent problems such as false positives, larger inserts, or missed large deletions, all detected variants were manually curated by inspecting the regions and the aligned reads. In addition, we performed independent analyses based on depth of coverage to detect duplications and deletions not identified by Delly. Calling duplications/deletions at genomic regions with variable coverage is a widely accepted methodology (Boeva et al. 2010). For every *C. parapsilosis* CDC317 gene, we computed the number of aligned reads per kilobase of coding sequence per million of aligned reads (RPKM). For any given experiment, RPKM is expected to be constant if there is no duplication/deletion. We defined a gene duplication in a given strain if it displayed a log 2 ratio of observed versus expected RPKM >0.75. Similarly, we called putative deletions if log 2 ratio was smaller than −0.75. Ratios not only enable the detection of duplication and deletions but also inform about the number of copies that have been gained or lost in each event. Finally, we used split reads as an additional line of evidence. For this, we split in two and realigned those reads that had been aligned over less than 90% of their length. Alignments created in this way were often flanking putative structural variants (supplementary fig. S4, Supplementary Material online). All detected variants were manually curated and some were validated experimentally to ensure that our findings are free of biases coming from library preparation, sequencing, or alignment (see below for details of experimental validation). Sequences selected for validation were those flanked by paired-end reads (deletions 1–20), as this guarantees linearity of the chromosomes and therefore enable PCR validations. For the remaining 15 deletions we are not sure about linearity and they me represent translocations not suitable to the used PCR validation. In addition, we generated genome graphs for all chromosomes illustrating CNV and homozygous and heterozygous mutation densities (supplementary file S3, Supplementary Material online). CNVs in the ARR3 cluster region were also detected using this procedure in 59 *C. albicans* strains publicly available in European Nucleotide Archive database as of October 2012 (see supplementary table S5, Supplementary Material online).

### PCR Amplification

PCR was carried out using Fermentas DreamTaqTM DNA polimerase kit. A single reaction contained 1 × DreamTaq buffer (supplied with 20 mM MgCl_2_), 0.25–0.25 µM forward and reverse primers, 0.2–0.2 mM dNTPs, 20–50 ng genomic template DNA, and 1 U DreamTaq DNA polymerase in a final volume of 20 µl. This reaction mix was incubated under the following conditions: 3 min of predenaturation at 94 °C was followed by 35 cycles of 15 s at 94 °C, 15 s at 56–60 °C, and 15 s to 1:40 min at 72 °C, and a final extension at 72 °C for 3 min. All the primers used in this study are listed in supplementary table S1, Supplementary Material online.

### Gel Electrophoresis

Most of the amplicons were analyzed on 1% agarose gel prepared with 1 × TAE containing 10 µl/100 ml ethidium bromide. Shorter deletions required 2% (Deletion 16 and Deletion 17) and 3% (Deletion 18, Deletion 19, and Deletion 20) agarose gels. The results were visualized and documented using UVP Bio-Doc-It System.

### Isolation of DNA Fragments for Sanger Sequencing

PCR products were isolated from agarose gel using Geneaid Gel/PCR DNA Fragments Extraction Kit according to the manufacturer’s instructions. The quality and quantity of the isolated DNA fragments were checked by gel electrophoresis.

### PCR Amplification of DIG-dUTP Labeled Probes for Southern Hybridization

For DIG labeling, the DreamTaqTM DNA polymerase kit (Fermentas) was used based on the protocol provided by the manufacturer. The dNTP mix was prepared with dNTPs (Fermentas) supplemented with Digoxigenin-11-dUTP (Roche). The PCR program consisted of the following steps: 3 min of predenaturation at 94 °C was followed by 35 cycles of 15 s at 94 °C, 15 s at 52–57 °C, and 1:20 min at 72 °C, and a final extension at 72 °C for 3 min. The sequences of the primers and the precise annealing temperatures with the volume of Digoxygenin-dUTP (DIG-dUTP) used in each reaction are listed in supplementary table S1, Supplementary Material online.

### Southern Hybridization

Genomic DNA was isolated as previously described and digested overnight with the following restriction enzymes (Fermentas): KpnI-SacI (Deletion 3), EcoRI (Deletion 5), HindIII (Deletion 6), according to the manufacturer’s instructions. DNA was precipitated with isopropanol and washed with 70% ethanol. Restricted DNA was separated on a 0.8% agarose gel along with DIG-labeled DNA Molecular Weight Marker VII (Roche). Fragments were transferred to Amersham HybondTM-N (GE Healthcare) filter and covalently linked by UV exposition. The subsequent steps were based on the Southern Blotting protocol of the Gottschling Lab (Fred Hutchinson Cancer Research Center) (http://labs.fhcrc.org/gottschling/General%20Protocols/southerns.html, last accessed December 2, 2013). Hybridization was performed overnight at 65 °C with the DIG-labeled PCR products described previously. To make the fragments visible, the samples were mixed with antidigoxigenin-AP Fab fragments (Roche) and then nitro blue tetrazolium chloride/5-bromo-4-chloro-3-indol-phospate (NBT/BCIP) Stock Solution (Roche) according to the manufacturer’s instructions.

### Phylogenetic Analyses

A phylogenetic tree of the four *C. parapsilosis* strains was reconstructed by concatenating all sites for the CDC317 genome, for which there was no deletion in any of the other strains. The corresponding sequence for the remaining three strains was filled in based on the information from the SNPs (i.e., the CDC317 sequence was altered if a SNP was detected at that site). To root this phylogeny, we created a second supermatrix of 92,754 bases by concatenating all SNPs and 50 bases surrounding the region and then adding the orthologous sequence from *C. orthopsilosis* (Riccombeni et al. 2012). Phylogenetic reconstructions were performed using RAxML (Stamatakis et al. 2005) version 7.28 using general time reversible (GTR) substitution matrix with 100 bootstrap replicates. Maximum likelihood phylogenetic analysis of ALS proteins was performed using the PhylomeDB pipeline described elsewhere (Huerta-Cepas et al. 2011).

## Results and Discussion

### Genome Sequencing and Analysis

We obtained the sequences of a clinical *C. parapsilosis* isolate from human blood from Germany (GA1) as well as two environmental *C. parapsilosis* strains isolated from healthy human skin in USA (CBS6318) and from an olive tree in Italy (CBS1954). In all cases, the strains were sequenced using a paired-end reads strategy (see Materials and Methods) to an estimated coverage ranging from 240 to 510 fold (table 1). These sequences were assembled de novo and annotated for protein-coding genes, and both the raw reads and annotated contigs have been deposited in EBI-ENA database (ERP002387). In addition, we used the available Sanger-based sequence of the *C. parapsilosis* CDC317 clinical strain, which was initially isolated from the hands of a hospital worker, who was the source for an outbreak of infection in a Mississippi community hospital in 2001 (Kuhn et al. 2004; Butler et al. 2009; Guida et al. 2011). This strain was used as a reference to detect various types of genomic variation including 1) SNPs, 2) CNVs, and 3) genomic rearrangements. As we will outline later in more detail, all lines of evidence show a relatively large degree of genomic divergence within the *C. parapsilosis* species, whose patterns of variation—rather than the degree of variation—are not compatible with the proposed clonal structure of the species.

### Genomic Content: Large Degree of Variability among ALS Genes

**Fig. 1.—:**
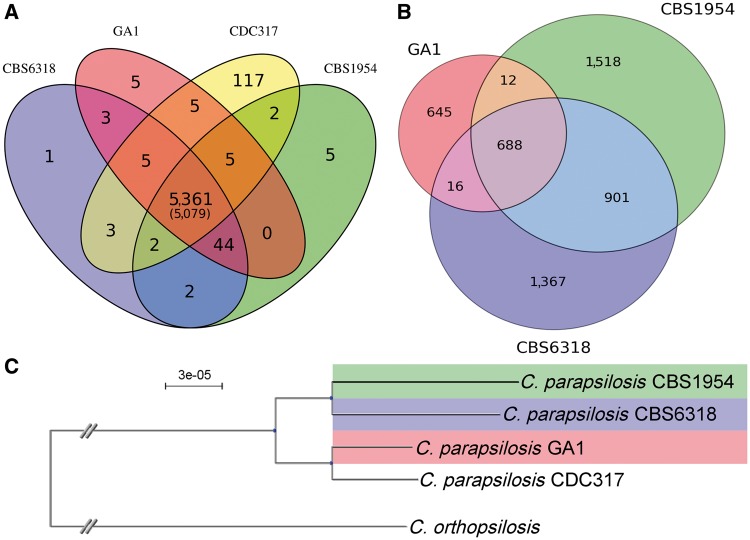
(*A*) Number of orthologs groups—that is, including orthologous genes and their recently duplicated paralogs—among the three newly sequenced *C. parapsilosis* strains; In brackets is indicated the orthologous groups that are single copy across all strains. (*B*) Shared SNPs using CDC317 as a reference for the three newly sequenced strains. (*C*) SNP-based phylogeny of four *C. parapsilosis* strains, using the closely related *C. orthopsilosis* as an outgroup.

As expected, the three strains sequenced in the context of this project showed a gene content that is largely similar to that of the reference strain (fig. 1*A*). Most observed differences concerned uncharacterized genes that largely correspond to potential differences in the annotation due to assembly fragmentation (see Materials and Methods). We found that, in or conditions, most of the annotated genes (∼91%) were expressed at significant levels (10 RPKM or more), and virtually all (99.9%) at detectable levels (1 RPKM or more). The use of different experimental conditions and strains prevent us to make meaningful comparisons to earlier expression data (Guida et al. 2011), but we could confirm that 98% of the genes expressed in our strains and conditions are also expressed in any of the replicas in the other study. Among our strains we detected 46, and 76 genes whose expression is only detected in environmental or clinical strains, respectively (supplementary table S6, Supplementary Material online). The functional gene ontology term “ion binding” was found to be enriched among genes expressed solely in clinical strains. Notably, we found that all strains contained the two bacterial-derived genes (Proline racemase, CPAR2_804470 and Phenazine F [PhzF], CPAR2_407660) previously detected (Fitzpatrick et al. 2008), indicating that this horizontal acquisition predated the divergence of the strains included here, and have been retained since then. These sequences were found to be expressed in all strains and be entirely conserved at the sequence level except for one synonymous SNP in GA1 for the Proline racemase gene. In addition, several CNVs affecting protein-coding genes were highly supported (see analyses later).

We focused on families that have been associated with virulence in *C. parapsilosis* (Butler et al. 2009). Supplementary table S7, Supplementary Material online, lists the gene copy number in virulence-related genes among the considered strains. Most of the families have similar numbers but small differences can be found among lysophospholipases, efflux pumps, and peptidases. Larger differences, however, were found in the ALS gene family, which encodes large cell-surface glycoproteins that function in host–pathogen interactions (Hoyer et al. 2001). In *C. albicans*, the family includes eight different genes (ALS1-7 and ALS9; Hoyer et al. 2008), each with an extensive degree of allelic variability, sometimes within a given strain or across the wider population of *C. albicans* (Zhang et al. 2003; Oh et al. 2005). Although some of the ALS family members—such as Als1p, Als3p, and Als5p—are better characterized, the cellular distribution and function of others remain to be determined. Although they share a similar structure, the members of the ALS family in *C. albicans* appear to differ from each other in their expression patterns and cellular functions. Different studies on Als1p and Als3p in *C. albicans* have shown their involvement in pathogenesis mediated through processes such as, among others, endothelial cell adhesion, biofilm formation (Hoyer et al. 2008), and invasion (Phan et al. 2007). ALS genes have also been shown to exist in other *Candida* species. Comparative genomic studies have revealed that most of the pathogenic *Candida* species, including *C. parapsilosis, C. tropicalis*, and *C. dubliniensis*, possess multiple ALS genes (Butler et al. 2009; Jackson et al. 2009), whereas the genome of *Saccharomyces* species lack any ALS ortholog. We determined the presence and distribution of ALS genes in the analyzed strains of *C. parapsilosis*. Surprisingly, we found a large intraspecies variability in terms of ALS family size. Different strains code a variable number of ALS genes, ranging from a single member (GA1, CBS1954), to three (CBS6318) and five (CDC317) members, all genes were found to be expressed. Closer inspection of the assemblies revealed that four ALS genes (ALS1, ALS3–ALS5) have been lost due to a single deletion event specific to GA1, and that CBS1954 lost the ALS2 gene due to a ∼400 bp deletion (see supplementary fig. S1, Supplementary Material online).

**Fig. 2.—:**
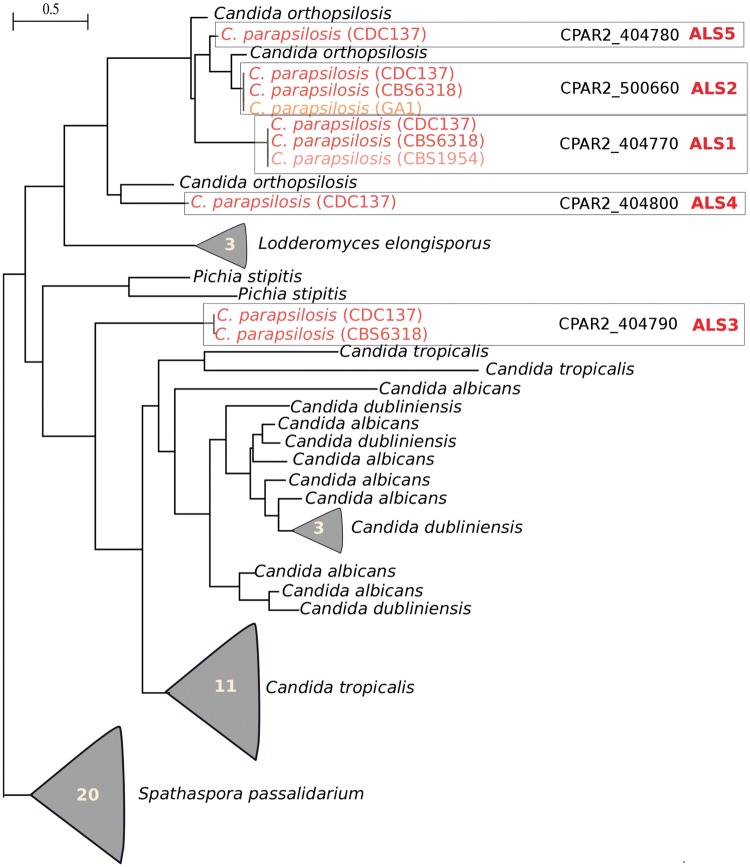
Maximum likelihood phylogenetic tree representing the evolutionary relationships among ALS family members in the four *C. parapsilosis* strains (marked in red/brown) and in other related *Candida* species (black). For simplicity, some clades of intraspecific paralogs have been collapsed indicating the number of sequences involved. The complete tree, including labeled nodes and sequence names, is available in newick format as supplementary file S5 (Supplementary Material online). Naming of ALS clades in *C. parapsilosis* species correspond to CDC317 genes.

Interestingly, the phylogeny of this family of genes across several related *Candida* species reveals an intricate story of expansions and losses (fig. 2), not only among the *parapsilosis* clade but also in related *Candida* species. This phylogenetic pattern is reminiscent of past events of gene conversion. To assess this possibility, we scanned for signatures of recombination in the relevant genomic regions encoding for ALS family members using nine different methods (see Materials and Methods). In *C. parapsilosis,* we found two strongly supported recombinations (supported by 7 out of 9 methods) comprising 760 bases in CPAR2_404800 and 280 bases in CPAR2_404780 (supplementary fig. S2, Supplementary Material online). Five additional potential recombination events were supported by —four to six methods (supplementary table S2, Supplementary Material online). Similar results were obtained in other *Candida* species. We found 13 likely recombination events in 5 out of 7 ALS genes in *C. albicans*, 19 possible recombination events in 8 out of 13 ALS genes in *C. tropicalis*, 19 possible recombination events in 5 out of 6 ALS genes in *C. dubliniensis;* and 6 possible recombination events in 3 out of 5 ALS gene in *Lodderomyces elongisporus* (supplementary table S2, Supplementary Material online). In contrast, the *Spathaspora passalidarum* genome encodes 20 ALS genes, but we found signs of only four possible recombination events in this species. For all species analyzed, most of the genes for which we detected recombination are placed in genomic proximity. This is consistent with the observed inverse relationship between the chromosomal distance of the homologous regions and the probability of recombination (Schildkraut et al. 2005). Notably, one of the clustered ALS genes in *C. parapsilosis* (CPAR2_404790) is apparently devoid of gene conversion footprints and seems to have diverged beyond the level of similarity necessary to undergo effective recombination with its neighboring homologs (39–59% identity at the nucleotide level, as compared to 49–74% in the other pairs). Altogether, these findings point to a highly dynamic state of ALS family within the *Candida* clade and that gene conversion has had an important role in shaping it.

### Nonhomogeneus Patterns of Single Nucleotide Variations Suggest the Presence of Recombination

We used a read mapping strategy to detect high-confidence SNPs of the different strains with respect to the reference genome CDC317 (see Materials and Methods). As previously reported, *C. parapsilosis* is highly homozygous (Butler et al. 2009) (see table 1). Overall, we detected 5,147, highly supported single nucleotide differences (SNPs) between any of our three sequenced strains and the reference, of which 1,361, 2,972, and 3,119 are present in GA1, CBS6318, and CBS1954, respectively. Out of these, 688 SNPs are shared among all three sequenced strains, and 901 are shared between CBS1954 and CBS6318, whereas GA1 shares only 12 and 16 mutations with CBS1954 and CBS6318, respectively (fig. 1*B*). Thus, the sequenced strains cluster by their isolation context (environmental/clinical) rather than by its geographical origin (USA/Europe). We confirmed this association with a phylogenetic analysis based on SNP data (Fig. 1*C*). This result is in line, for the shared strains, with a previous phylogenetic reconstruction based on mitochondrial genome sequences (Valach et al. 2012).

Overall, the average SNP density was 0.42 SNPs/kb or one SNP every 2,376 bp. This corresponds to a nucleotide diversity (π, defined as the average number of pairwise nucleotide differences per site in sampled DNA sequences [Nei and Li 1979]) of 0.42 × 10^−3^. If the strains are taken separately, the mutation density is in the range of 0.10–0.25 per 1 kb, corresponding to one SNP every 10,517 bp (GA1), 4,306 bp (CBS6318), or 4,024 bp (CBS1954). Importantly, the identified SNPs are not homogeneously distributed along the chromosomes, and we could identify 166 regions in which the three consecutive mutation occur within 200 bases (density is larger than 5 SNPs/kb). These regions represent 39.8 kb (0.3%) of the *C. parapsilosis* genome and comprise 713 (13.8%) of the identified SNPs. When analyzing strains separately, 34 SNPs are grouped in 10 such clusters in GA1, 341 SNPs in 78 clusters in CBS6318, and 341 SNPs in 88 clusters in CBS1954. If the SNPs are considered to be the result of independent mutation events (as would be expected from all variation resulting from vertical descent from an ancestral clone), and given the very low density of SNPs, then observing three SNPs in a 200 bp fragment is a very unlikely event [*p*(*x* > = 3) = 2 × 10^−^^5^, as computed using a Poisson distribution for CBS1954, the strain with the highest SNP density]. Consequently, the expected number of such hotspots in a 13 Mb genome would be much lower than the observed number of clusters (1 vs. 88 for CBS1954). Thus, the most plausible scenario is that a significant number of such hotspots originated through a mechanism other than spontaneous point mutations.

A possible mechanism for the origin of this pattern of SNP clustering is by means of recombination among strains. To test this possibility, we scanned genome alignments to detect such events (see Materials and Methods). Twenty possible recombination events were identified involving five chromosomes: 3 events in HE605203, 5 in HE605204, 3 in HE605206, 2 in HE605207, and 7 in HE605209 (see supplementary table S2, Supplementary Material online). Notably, 51 of 166 detected variation hotspots overlap with 12 of 20 detected recombination events, indicating that, at least in part, variation hotspots could have been created by recombination among different strains. As we report in the next section, the existence of recombination is also independently supported by the presence of larger, CNVs with identical boundaries that are shared among strains that are not monophyletic. To our knowledge, this is the first time that evidence for interstrain recombination has been identified in *C. parapsilosis*. No recombination was found in the remaining nuclear chromosomes or, in agreement with an earlier analysis (Valach et al. 2012), in the mitochondrial chromosome.

### CNV Analyses Identify Independent Expansions of an Arsenite Transporter Homolog

We predicted CNVs based on a depth of coverage approach (Yoon et al. 2009) by mapping the reads from the sequenced strains onto the reference. Regions with significantly higher or lower coverage (log 2 > 0.75 or log 2 < –0.75) and/or having inconsistent read pairing were manually inspected to discard possible assembly or mapping artifacts (see Materials and Methods). Altogether, 40 CNVs were detected in the three newly sequenced *C. parapsilosis* strains (supplementary table S3, Supplementary Material online). We selected 20 events for experimental validation and all of them could be confirmed (see Materials and Methods, and Supplementary file S4, Supplementary Material online). Thirty-five CNVs involved deletions with respect to the reference strain. The size of these deletions ranges from 17 bases to 23 kb, with the majority of deletions being around 2 kb. Some deletions are present in more than one strain but most are strain specific (supplementary table S3, Supplementary Material online). Providing additional support for the idea of recombination among *C. parapsilosis* strains, closer inspection of some of the shared deletions revealed identical boundaries in various strains. For instance, the experimentally validated deletion DEL 15 (supplementary table S3, Supplementary Material online) is shared by the two European strains (GA1, CBS1954) that are not closely related according to phylogenetic and SNP data (see earlier).

**Fig. 3.—:**
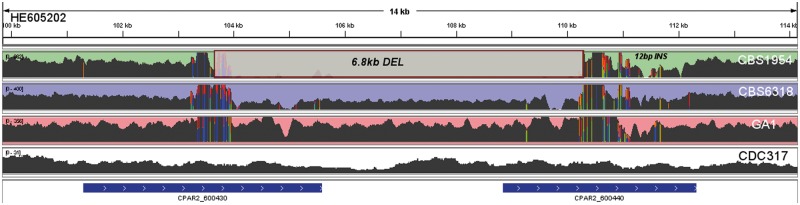
Schematic view of the region surrounding a CBS1954-specific deletion of 6.8 kb (DEL 31). Density of mapped reads along the coordinates of the reference strain (CDC317) is indicated. An unmapped region of 6.8 kb is identified as a deletion in CBS1954 only. This deletion is predicted to have originated an in-frame fusion of two genes CPAR2_600430 and CPAR2_600440.

Notably, most of the deletions (31) affect protein-coding genes and 18 are predicted to have resulted in gene fusions. Such fused genes are in-frame and were expressed in the tested conditions. For instance, a deletion of 6.8 kb (DEL 31) in a contig homologous to Chromosome HE605202 in CBS1954 (HE605206:1,156,870–1,158,502) in CBS1954 has likely resulted in a fusion of two genes, CPAR2_600430 and CPAR2_600440 (fig. 3). The upstream gene codes for a protein containing a “hyphally regulated cell wall protein N-terminal domain” (PF11765) and a region with PT repeats, whereas the downstream gene has no annotated domain. A closer analysis of the sequences of the fused genes revealed that they share two relatively long stretches (0.5 kb) of high nucleotide sequence identity (92%), which suggests recombination as the possible mechanism creating this fusion. Interestingly, DNA direct repeats, ranging from 8 to 835 bp of length, were found flanking all identified deletions. This points to the possibility that the detected variants were introduced by DNA double-strand breaks followed by single strand annealing repair (Ivanov et al. 1996). In ten cases, we confirmed the direct involvement of repeats by identifying the exact breakpoints through Sanger sequencing. In the remaining ten cases, Sanger reads were not of sufficient length to validate our hypothesis, but these cases are nevertheless supported by Illumina split-read mapping (supplementary table S3 and fig. S4, Supplementary Material online, for split-read example).

We found five duplication events, all involving protein-coding genes (see supplementary table S3, Supplementary Material online). In line with the previously described examples of recombination footprints, one duplication event (DUP 4) is shared by the same two European strains (GA1, CBS1954) that share DEL 15 and also in this case present identical boundaries in the two genomes. Other shared duplications seem to have originated in independent events.

**Fig. 4.—:**
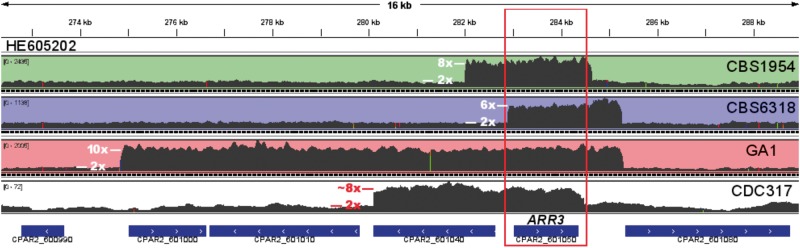
CNVs involving a homolog of ARR3 in the four *C. parapsilosis* strains examined. Data for the reference strain CDC317 was obtained by remapping the raw reads of that genomic project. Excess of coverage in a discrete genomic region indicates the presence of a higher number of copies. Although the region coding for ARR3 is included in the CNVs detected in all strains, the clearly distinct boundaries of the duplicated blocks clearly indicate that they originate from independent duplication events. Inferred number of copies based on depth of coverage is indicated.

Remarkably, the three *C. parapsilosis* strains sequenced in this study contain various copies of the gene ARR3 (eight copies in CBS1954, six in CBS6318, and ten in GA1). This seems to be also the case in the reference CDC317 (eight copies), as confirmed by remapping the raw reads of the sequenced project. Note that the current CDC317 assembly has collapsed these copies into a single copy, as it happens with other repeated regions of high similarity (e.g., rRNA gene cluster). Notably, the four strains differ in the number of duplication events, their length and coordinates of the repeated segment around the ARR3 gene (fig. 4, DUP 5 in supplementary table S3, Supplementary Material online), suggesting that the duplications are independent events. Finally, the presence of tail-to-tail (← →), instead of expected head-to-head (→ ←), orientation of paired reads at duplicated region ends in the three newly sequenced strains indicates that the repeated copies are organized in tandem. By split-read mapping, we could also confirm the presence of this repeats in tandem in CDC317. Our expression data showed that expansion of the ARR3 cluster indeed leads to higher expression of this gene when compared with nonduplicated ones in the close vicinity. Altogether, these findings suggest that the amplification of ARR3 gene may have been driven by selection. ARR3 is homologous to an arsenite transporter of the plasma membrane in *S. cerevisiae,* required for resistance to arsenic compounds. Considering the generally low levels of arsenic or other metalloids in the human body and the fact that the CNVs involving ARR3 are also found in environmental isolates, it is likely that the selective pressure for this expansion is present in the environment rather than in the human host. Consistent with an environmental selection, the ARR3 gene was found to be expressed at higher levels in the two environmental strains (77–114 RPKM) when compared with the two clinical isolates (12–54 RPKM) despite the fact that identical conditions for growth were used. Finally, supporting the idea that ARR3 expansion did not result from adaptation to the human host, we did not find any such expansion in 59 *C. albicans* strains with raw genome data deposited in the European Nucleotide Archive (see supplementary table S5, Supplementary Material online). This reinforces the view that *C. parapsilosis*, in contrast to other *Candida* species such as *C. albicans,* is not exclusively adapted to a mammalian host but rather is naturally adapted to other environments where it can be often found. In addition, the observation that the two clinical isolates carry different expansions likely selected in the natural environment indicates that they do not derive from a single clone that expanded in a clinical context but rather from two independent natural populations.

### Concluding Remarks

We have presented the first genome-wide genetic variability analysis within *C. parapsilosis,* an important fungal pathogen of increasing incidence. Previous analyses of genome variability within this species were based on a few marker genes and gave rise to the hypotheses that *C. parapsilosis* clinical isolates were the result of a recent clonal expansion and that they lack the ability to recombine due to the degeneration of some mating-type genes. Our results based on the higher resolution provided by massive sequencing techniques, however, point to an unexpectedly high level of intraspecies variability in relevant gene families involved in virulence, such as the ALS family of adhesins. We find evidence for intrachromosomal recombination and identify the presence of tandem repeats as a mechanism to generate CNV among strains, involving the deletion of genes and also the creation of potential new, fused genes. In addition, we found that genomic regions of high variation are likely the result of recombination events among different strains, providing the first such evidence in this species. As independent evidence for recombination among clinical and environmental lineages, we report the existence of large deletion and duplication events with identical boundaries in strains that are not monophyletic. Such patterns critically challenge the scenario in which all clinical isolates derived vertically from a single virulent clone. Finally, we show the existence of independent expansions of an arsenite transporter cluster in all examined strains. This pattern is indicative of selection in the environment and provides further support against proposed clonal expansion of the species in the human host. There is very little information regarding the natural habitat of *C*. *parapsilosis*. This species has been isolated from nonhuman sources, including feces of domesticated animals, from insects, from soil or even from marine environments (Van Uden et al. 1958; Fell et al. 1960; Carruba et al. 1991). The availability of genome sequences from clinical and environmental isolates may help future studies, aiming to unveil the natural habitats of *C. parapsilosis* and its adaptation to the human host.

## Supplementary Material

Supplementary files S1–S5 are available at *Genome Biology and Evolution* online (http://www.gbe.oxfordjournals.org/).

Supplementary Data
