# Supplementary material for: Unexpected Genomic Variability in Clinical and Environmental Strains of the Pathogenic Yeast Candida parapsilosis
Source: Genome Biol Evol. 2013 Nov 20;5(12):2382–92. doi: 10.1093/gbe/evt185 (PMC3879973; doi:10.1093/gbe/evt185)
Supplement: Supplementary Data [file supp_5_12_2382__index.html]

Unexpected genomic variability in clinical and environmental strains of the pathogenic yeast Candida parapsilosis — Unexpected Genomic Variability in Clinical and Environmental Strains of the Pathogenic Yeast Candida parapsilosis — Supplementary Data 

# Unexpected Genomic Variability in Clinical and Environmental Strains of the Pathogenic Yeast *Candida parapsilosis*

## Supplementary Data

files

**Files in this Data Supplement:**

- Supplementary Data - pdf file
- Supplementary Data - pdf file
- Supplementary Data - pdf file
- Supplementary Data - xls file
- Supplementary Data - txt file
