# Supplementary material for: Unexpected Genomic Variability in Clinical and Environmental Strains of the Pathogenic Yeast Candida parapsilosis
Source: Genome Biol Evol. 2013 Nov 20;5(12):2382–92. doi: 10.1093/gbe/evt185 (PMC3879973; doi:10.1093/gbe/evt185)
Supplement: Supplementary Data [file supp_evt185_SuppFile1.pdf]

Supplementary file 1: Supplementary figures and tables

Figure S1: ALS gene family deletions......1
Figure S2: Recombinations among ALS genes......2
Figure S3: Example of structural variants supporting recombination hypothesis......3
Figure S4: Example of an alignment used to detect structural variants......4
Figure S5: Fusion of CPAR2_300120 and CPAR2_300110 as result of DEL25......5

Figure S1: ALS gene family deletions

Example is showing ALS2 deletion in CBS1954 (A) and deletion of ALS1, AlS5, ALS3 and ALS4 in GA1 (B). Deleted regions are marked with violet frames.

A. loss of ALS2 in CBS1954

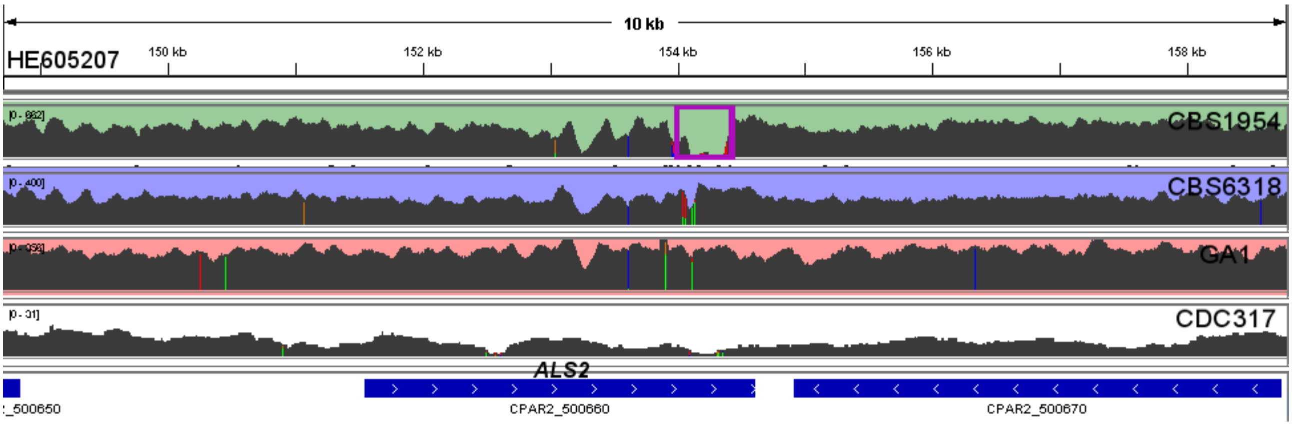

B. loss of ALS1, AlS5, ALS3 and ALS4 in GA1 due to DEL#1

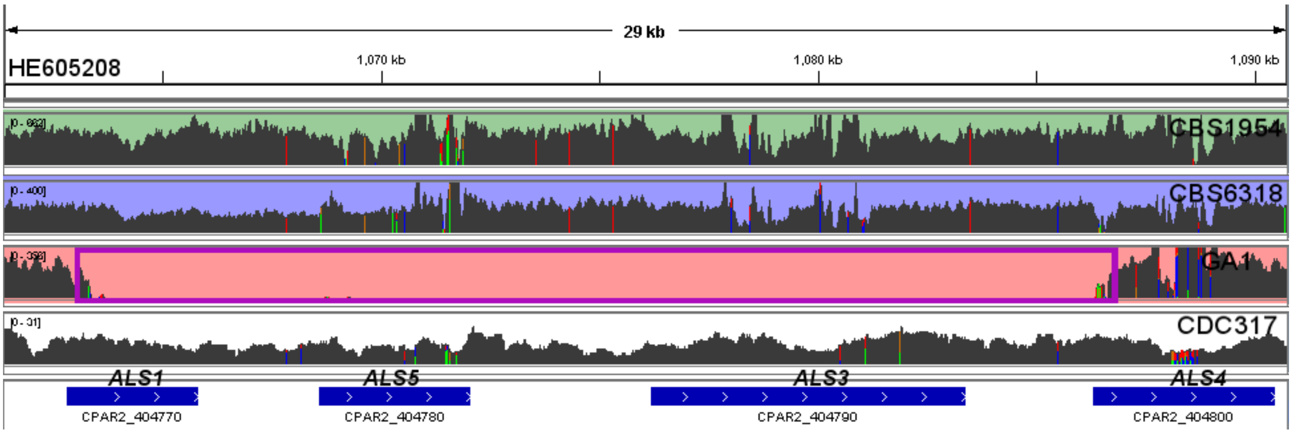

Figure S2: Recombinations among ALS genes

Five members of *Candida parapsilosis* ALS gene family were tested for recombination using RDP3. For each gene (long horizontal bars), we plotted regions that recombined (lighter blocks) together with potential parental donor sequence (shorter bars given below). Potential recombinations were found in four ALS genes: CPAR_40770, CPAR_40780, CPAR_40800 and CPAR_500660. CPAR_40790, the most diverged member of this family, is the only ALS gene for which we found no evidence of recombination. Detailed results for these recombinations can be found in Table S1.

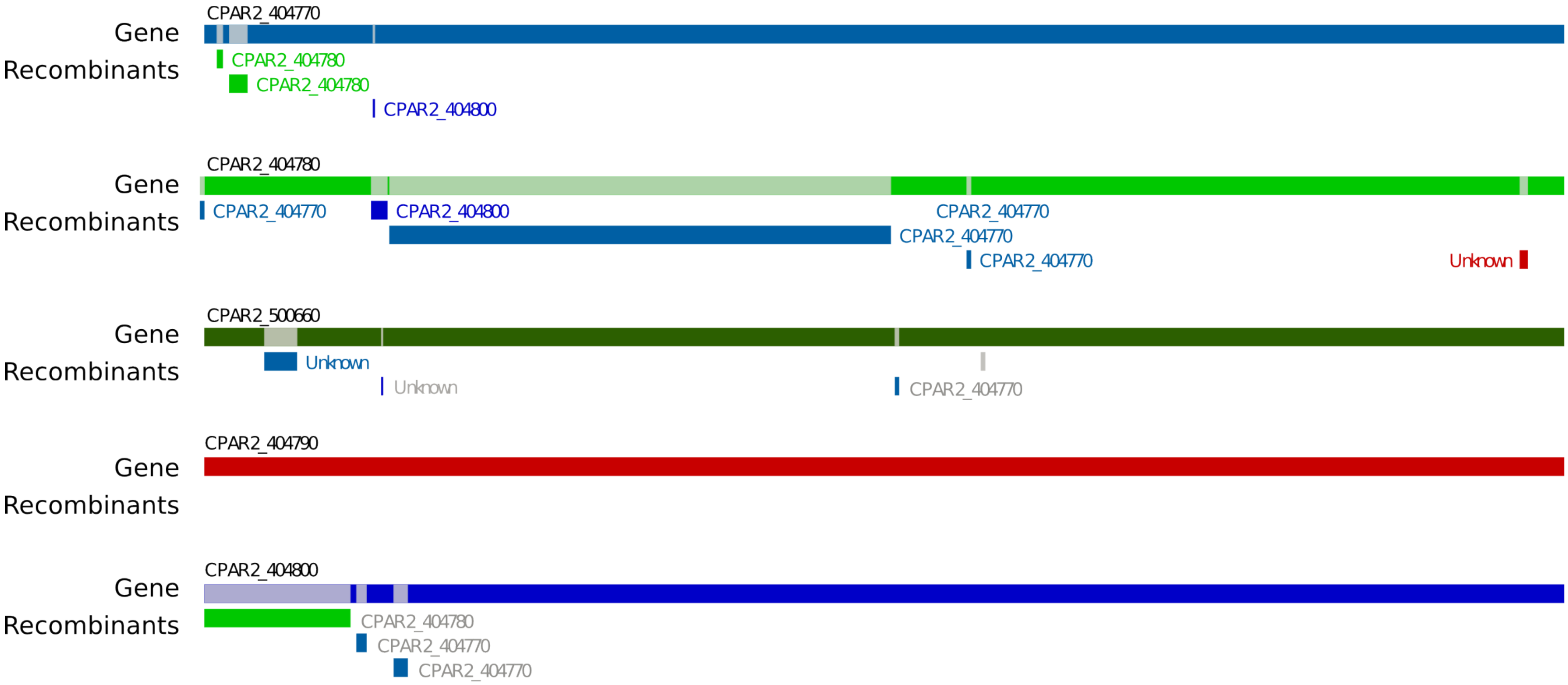

Figure S3: Example of structural variants supporting recombination hypothesis

Recombination between CBS1954 and GA1 (A) is supported by deletion 15 in chromosome HE605203 (B) and duplication 4 in chromosome HE605204 (marked by stars).
A) Strains tree is given together with table of deletions (blue) and duplications (red). For simplicity, only events affecting more than one strain are shown. Number of copies is provided for each event, if different than two (expected in diploid). For DEL20 two alternative deletion sizes are provided. Note, DEL6 and DUP5 are independent events in all strains (for details see Fig S4 and Fig 4).
B) DEL15 was called in CBS1954 and GA1 due to: i) elevated insert size of read pairs (marked in read) and ii) drop in depth of coverage. DNA direct repeat likely causing this deletion was identified. Finally, DEL15 was confirmed by split reads and PCR (see Supplementary file 3).

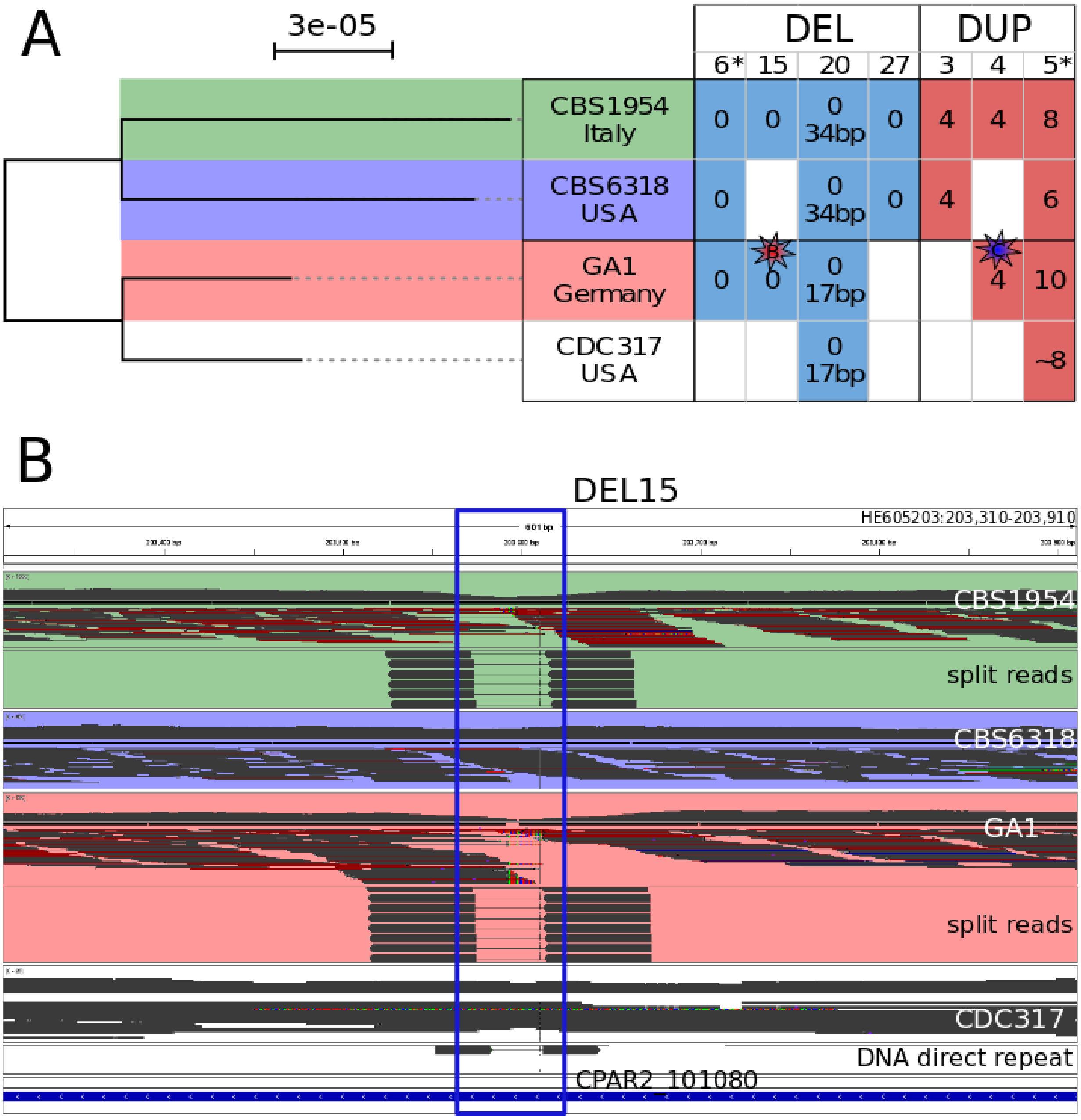

Figure S4: Example of an alignment used to detect structural variants

Example of an alignment used to detect deletions. DEL#6 (HE605206:1,156,841-1,158,435) is shown. Copy numbers were identified by means of: i) reduced depth of coverage ii) and/or significantly elevated insert size between paired-end reads. Subsequently, we have checked wether split-read mapping and DNA direct-repeats are present around detected deletions.

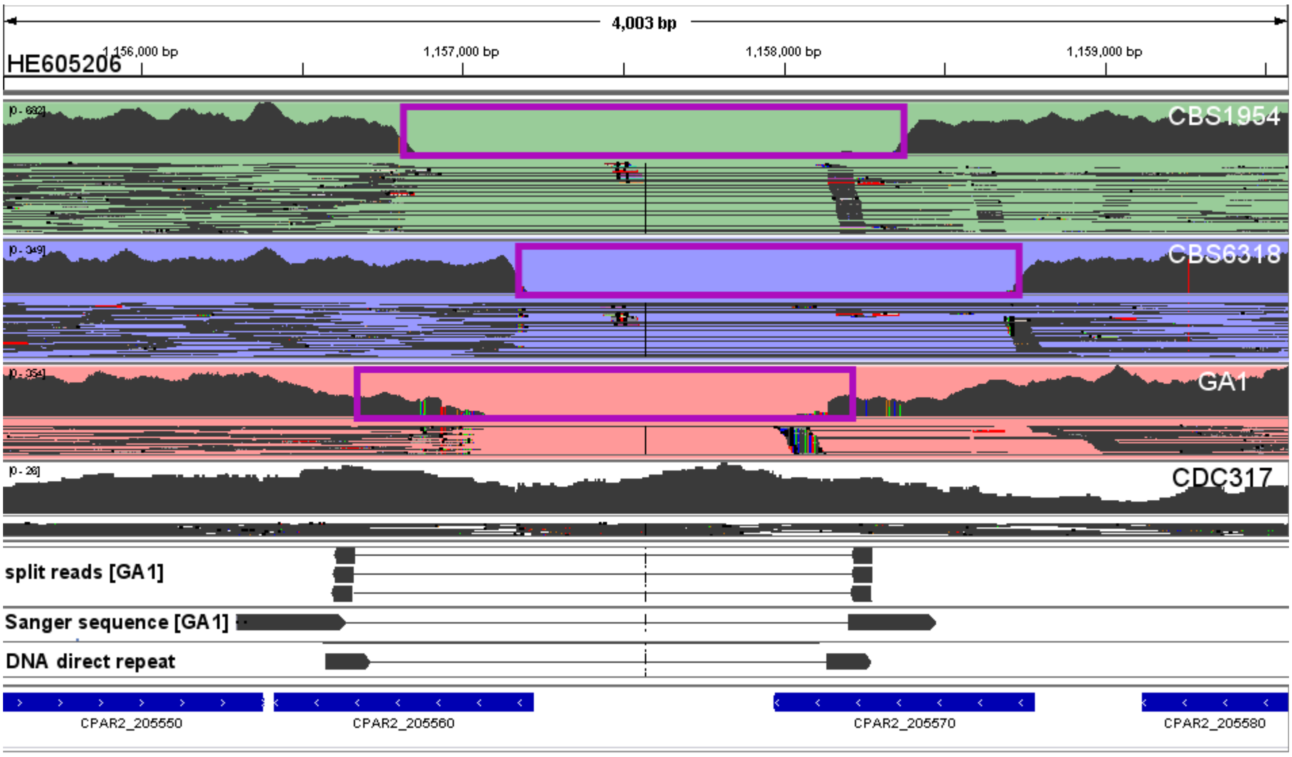

Figure S5: Fusion of CPAR2_300120 and CPAR2_300110 as result of DEL25

A. Gemome organisation around DEL25

Predicted genes together with reads coverage from genome and transcriptome are plotted for each strain. Two genes orthologous to CPAR2_300120 and CPAR2_300110 are predicted in CBS1954 and GA1. In contrast, in CBS6318, due to 4,174 bp deletion, there is only one gene predicted. Sequence of that gene correspond to CPAR2_300120 in N-terminal end and to CPAR2_300110 in C-terminal end (**B**). Central part is identical between these proteins and it corresponds to identical gene sequence where recombination took place. Resulting fused protein is slightly longer (1036aa) than both, CPAR2_300120 (924aa) and CPAR2_300110 (918aa). Protein domains were annotated by InterProScan5 (Hunter *et al*, 2011).

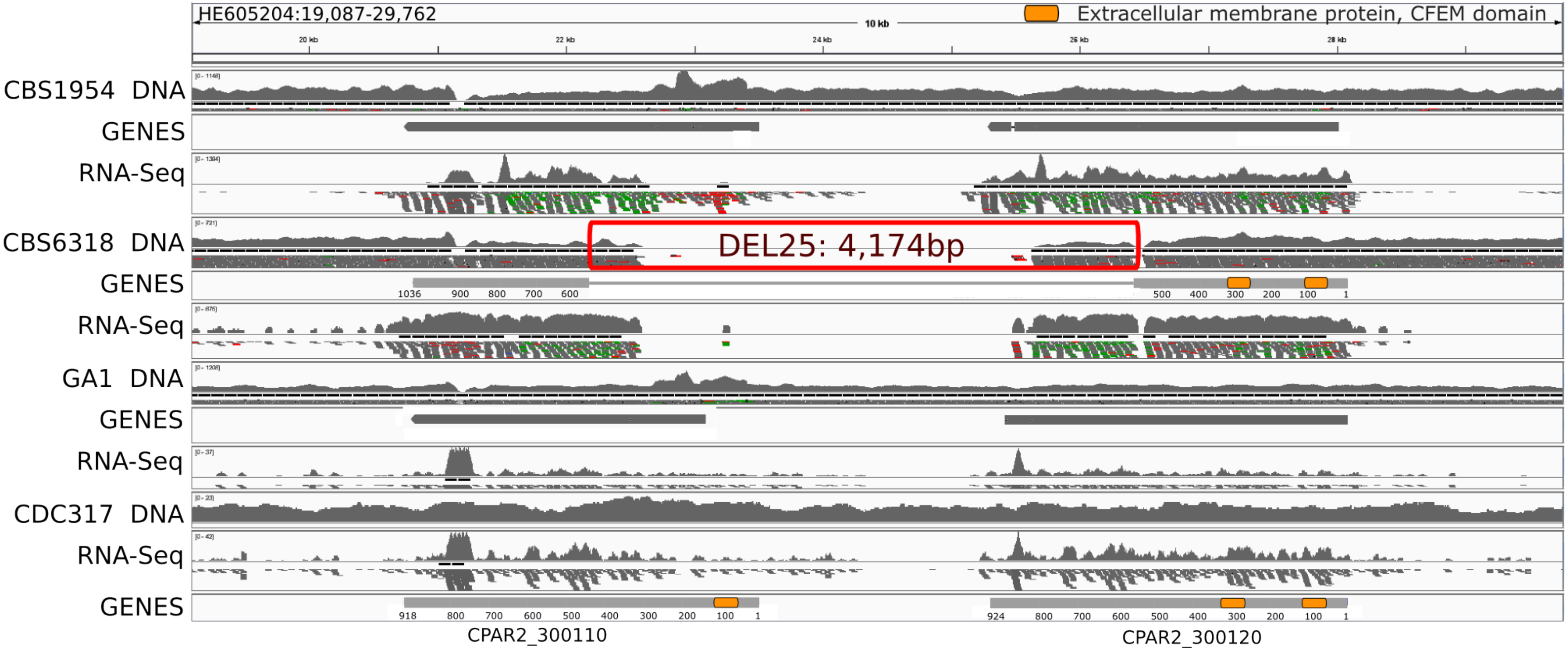

B. Multiple sequence alignment of CPAR2_300120, CPAR2_300110 and fused protein

Sequence shared by fused protein and CPAR2_300120 and/or CPAR2_300110 are in bold.

```
CPAR2_300110        MKYEFIYCLIAILSIAIAADEDSITTAPTTIVDNPYATYPSVPKTASINGFADRIYDLLP
CPAR2_300120        MKYTLVYSLASILSLVVAADEDSITTAPTT-TTNPYATYPSVAKTASINGFADRIYDQLP
fused_gene          MKYTLVYSLASILSLVVAADEDSITTAPTT-TTNPYATYPSVAKTASINGFADRIYDQLP
                    *** ::*.* :***:.:************* . *********.************** **

CPAR2_300110        ECAKPCMFQNTGVTPCPYWDTGCLCIMPMFQDKIGTCIAENCRGEDVLDARSLATSVCSI
CPAR2_300120        ECAQPCMFQNTGVTPCPYWDTGCLCIMPMFQDKIGKCIAENCRGEDVLDARSLATSICSS
fused_gene          ECAQPCMFQNTGVTPCPYWDTGCLCIMPMFQDKIGKCIAENCRGEDVLDARSLATSICSS
                    ***:*******************************.********************:**

CPAR2_300110        AGVWDPYWMPPASVTSMLEFAATAAPISSTVE---SSIEETTIEESSV--EESITEESTA
CPAR2_300120        AGVWEPYWMPPASVSSMLESAATAVPVTSTTEESSSTEESSSTEESSSTEESSSTEESSS
fused_gene          AGVWEPYWMPPASVSSMLESAATAVPVTSTTEESSSTEESSSTEESSSTEESSSTEESSS
                    ****:*********:**** ****.*::**.*   *: *.:: ****   *.* ****::

CPAR2_300110        EE---STIVEPTAEE-STAEESTAEESTAEESTIVE----------PTAEESSVVE----
CPAR2_300120        TEESSSTEVSSSAEETSSVEESSTVESSTEESSTAEESSTSSISQAPSSSSDSLITSAPS
fused_gene          TEESSSTEVSSSAEETSSVEESSTVESSTEESSTAEESSTSSISQAPSSSSDSLITSAPS
                     *   ** *..:*** *:.***:: **::***: .*          *::...*::

CPAR2_300110        ------------------------------PSVVESSVVESTVVEP--------------
CPAR2_300120        SSTTNPYATYPSVAKTASINGFADRIYDQLPECAQPCMFQNTGVTPCPYWDTGCLCIMPT
fused_gene          SSTTNPYATYPSVAKTASINGFADRIYDQLPECAQPCMFQNTGVTPCPYWDTGCLCIMPT
                                                  *. .:..:.:.* * *

CPAR2_300110        ------------------------------------------------------------
CPAR2_300120        FQGAVGKCIAENCRGEDVLDARSLATSICSSAGVWEPYWMPPASVSSMLESAATAVPITS
fused_gene          FQGAVGKCIAENCRGEDVLDARSLATSICSSAGVWEPYWMPPASVSSMLESAATAVPITS

CPAR2_300110        ------------TAEESTAEESTVVEPTAEESITEESTAEESTTEESTAEESTVMEPSAE
CPAR2_300120        SDDSNSQASNTESLSQSTDDTVSSADPSAESSADPSAESSAEPSAESSAEPSA--ESSAE
fused_gene          SDDSNSQASNTESLSQSTDDTVSSADPSAESSADPSAESSAEPSAESSAEPSA--ESSAE
                                : .:** :  : .:*:**.*   .: :. ..: **:** *:  *.***

CPAR2_300110        PSVESSAEPSASHGTTDSVFASSVESTLPTESVTPGESFVISALFNGEVTTFESNGTHIK
CPAR2_300120        PSVESSAEPSFSHGTTDSVSASSVESTLPTESVTPGESFVISALFNGEVTTFESNGTHIK
fused_gene          PSVESSAEPSFSHGTTDSVSASSVESTLPTESVTPGESFVISALFNGEVTTFESNGTHIK
                    ********** ******** ****************************************

CPAR2_300110        LYDGSEVQFRIVDGLLQVGESGYVHVGTVGELIVGDVDGATSGWGLENNRVTLTLLSATK
CPAR2_300120        LYDGSEVQFRIVDGLLQVGESGYVHVGTVGELIVGDVDGATSGWGLENNRVTLAPSSSAR
fused_gene          LYDGSEVQFRIVDGLLQVGESGYVHVGTVGELIVGDVDGATSGWGLENNRVTLTLLSATK
                    *****************************************************:  *::.

CPAR2_300110        VTFSDNEVQFYACPAEVGYDVCVADGEGCVPIGAVAGPLSAPESSTIDEASVTPSASESG
CPAR2_300120        LMKRENEVQFYACPAEVGYDVCVADGEGCVPIGAVAGPLSAPESSTIDEASVTPSASESG
fused_gene          VTFSDNEVQFYACPAEVGYDVCVADGEGCVPIGAVAGPLSAPESSTIDEASVTPSASESG
                    :   :*******************************************************

CPAR2_300110        DATGSSGVTETGASQSSDVSEPSVSHSSDVSHESYPSTSTVTSEITTTDEEGVASTLTTT
CPAR2_300120        DATGSSGVTETGASQSSDVSEPSVSHSSDVSHESYPSTSTVTSEITTTDEEGVASTLTTT
fused_gene          DATGSSGVTETGASQSSDVSEPSVSHSSDVSHESYPSTSTVTSEITTTDEEGVASTLTTT
                    ************************************************************
```

```
CPAR2_300110       IVVTKVSEYCAQESASSVQSEAHSAQTSAAAVQSTCIEKQQSIVTVIVSCESAISSLHSV
CPAR2_300120       IVVTKVSEYCAQESASSVQSEAHSAQTSAAAVQSTCIEKQQSIVTVIVSCESAISSLHSV
fused_gene         IVVTKVSEYCAQESASSVQSEAHSAQTSAAAVQSTCIEKQQSIVTVIVSCESAISSLHSV
                   ************************************************************

CPAR2_300110       KSSAQQVTKTEIVYSCESQISSLSSVEQQAHKTLEAVVSEYDSAVSVQKSAAEQQKSAAQ
CPAR2_300120       KSSAQQVTKTEIVYSCESQISSLSSVEQQAHKTLEAVVSEYDSAVSVQKSAAEQQKSAAQ
fused_gene         KSSAQQVTKTEIVYSCESQISSLSSVEQQAHKTLEAVVSEYDSAVSVQKSAAEQQKSAAQ
                   ************************************************************

CPAR2_300110       VQLSEAELQHSSEAVAHAQSAAAEAYTAAVEASQAASIASAAKETVAAVARTAPGAEETG
CPAR2_300120       VQLSEAELQHSSEAVAHAQSAAAEAYTAAVEASQAASIASAAKATVAAVARTAPGAEETG
fused_gene         VQLSEAELQHSSEAVAHAQSAAAEAYTAAVEASQAASIASAAKETVAAVARTAPGAEETG
                   ******************************************* ****************

CPAR2_300110       ETAPQGEGAGETAPQGEGAGETASQSEGAGETASQSEGAGEAAPQGEGAGEAAPQGEGAG
CPAR2_300120       ETAPQGEGAGEAA------------------------------PQGEGAGEAAPQG----
fused_gene         ETAPQGEGAGETA----------SQSEGAGETASQSEGAGEAAPQGEGAGEAAPQGXXXX
                   ***********:*                              *************

CPAR2_300110       EAAPQGEGAGEAAPQGEGAGEAAPQGEGAGEAAPQGEGAGEAAPQGEGAGEAAPQGEGAG
CPAR2_300120       ------------------------------------EGAGEAAPQDESEAAKAQ------
fused_gene         XXXXX--------XXXXXXXXXXXXXXXXXXXXXXXEGAGEAAPQGEGAGEAAPQGEGAG
                                                       *********.*. .  *

CPAR2_300110       ENQITQQGEGEEYVPQDVVQTLTISSTIYVTSGGESTTISSTGEIDNPTKSVGSANSIIS
CPAR2_300120       -----------------------------------------------------ASSSVSE
fused_gene         ENQITQQGEGEEYVPQDVVQTLTISSTIYVTSGGESTTISSTGEIDNPTKSVGSANSIIS
                                                                        ::.*: .

CPAR2_300110       IDETTQSAESINAGHKLSCGVYLSLMVTTSMFVLFSI
CPAR2_300120       TSNETQTIAAVNAGAK-SFGLSMSMMSTIVAFAALLM
fused_gene         IDETTQSAESINAGHKLSCGVYLSLMVTTSMFVLFSI
                    .: **:  ::*** * * *: :*:* *   *. : :
```
