# Supplementary material for: Unexpected Genomic Variability in Clinical and Environmental Strains of the Pathogenic Yeast Candida parapsilosis
Source: Genome Biol Evol. 2013 Nov 20;5(12):2382–92. doi: 10.1093/gbe/evt185 (PMC3879973; doi:10.1093/gbe/evt185)
Supplement: Supplementary Data [file supp_evt185_SuppFile3.pdf]

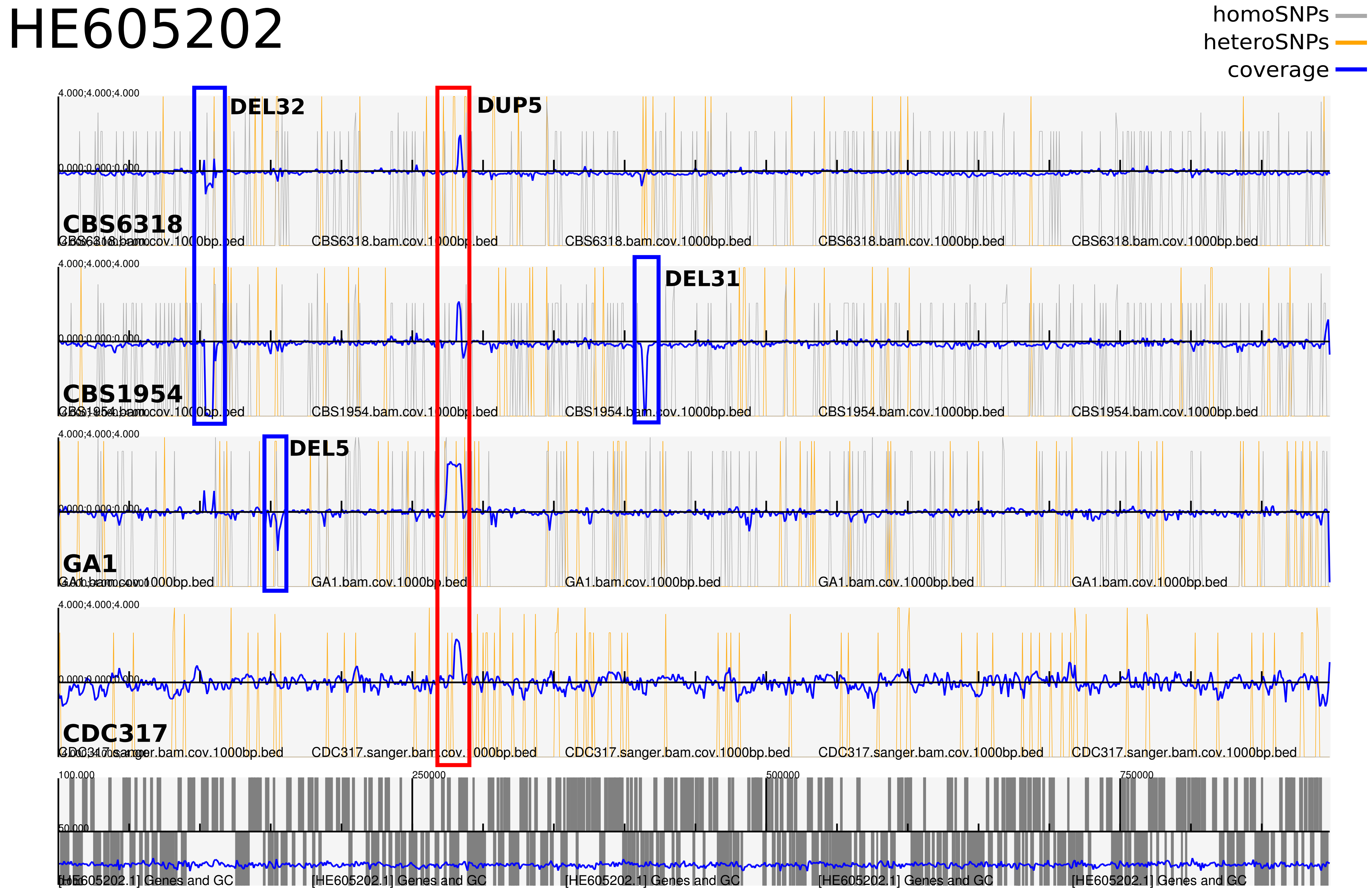

HE605202
homoSNPs
heteroSNPs
coverage
DEL32
DUP5
CBS6318
CBS6318.bam.cov.1000bp.bed
DEL31
CBS1954
CBS1954.bam.cov.1000bp.bed
DEL5
GA1
GA1.bam.cov.1000bp.bed
CDC317
CDC317.sanger.bam.cov.1000bp.bed
250000
500000
750000
[HE605202.1] Genes and GC

HE605203

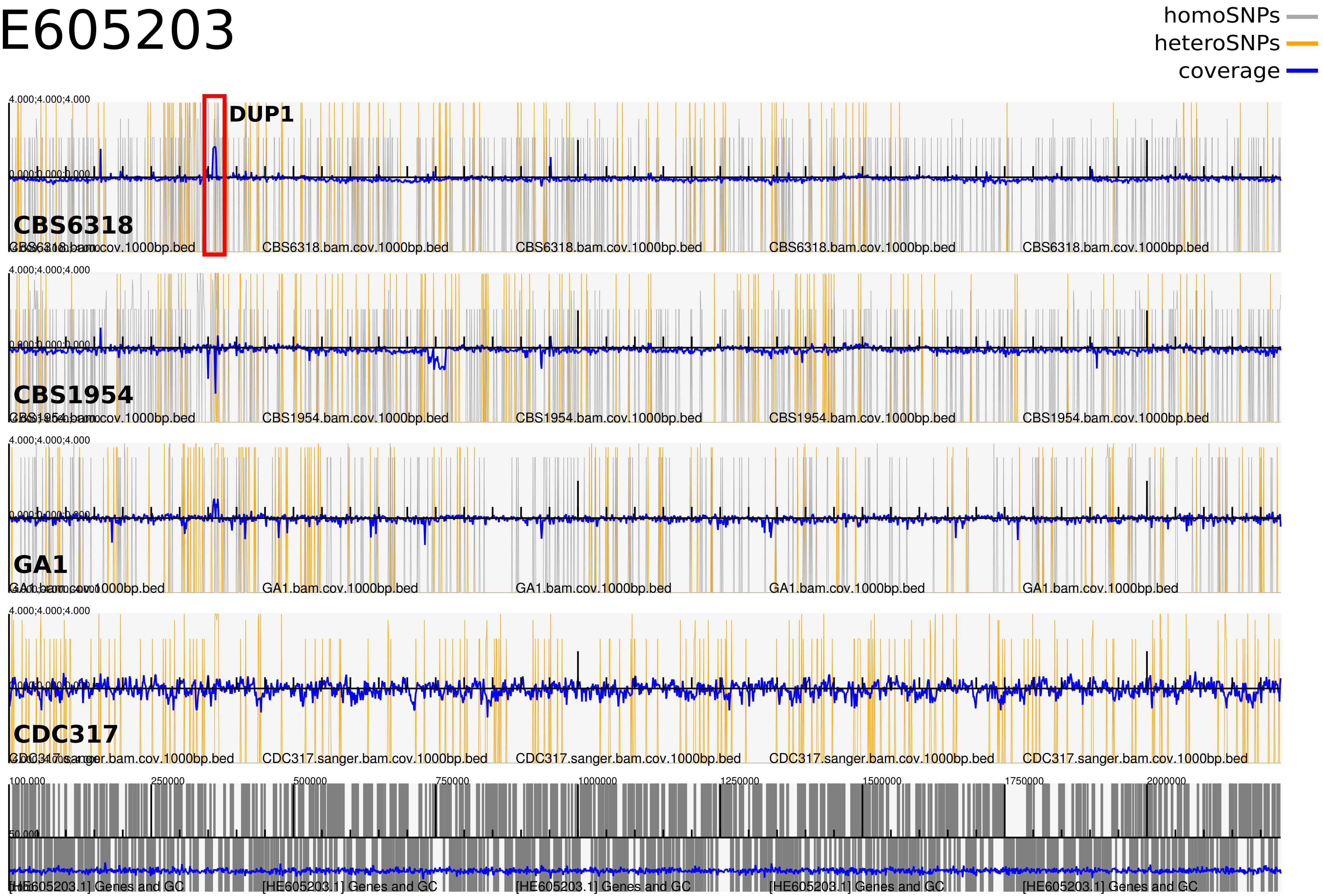
homoSNPs
heteroSNPs
coverage
DUP1
CBS6318
CBS1954
GA1
CDC317
CBS6318.bam.cov.1000bp.bed
CBS1954.bam.cov.1000bp.bed
GA1.bam.cov.1000bp.bed
CDC317.sanger.bam.cov.1000bp.bed
4.000;4.000;4.000
0.000;0.000;0.000
100.000
50.000
250000
500000
750000
1000000
1250000
1500000
1750000
2000000
[HE605203.1] Genes and GC

HE605204

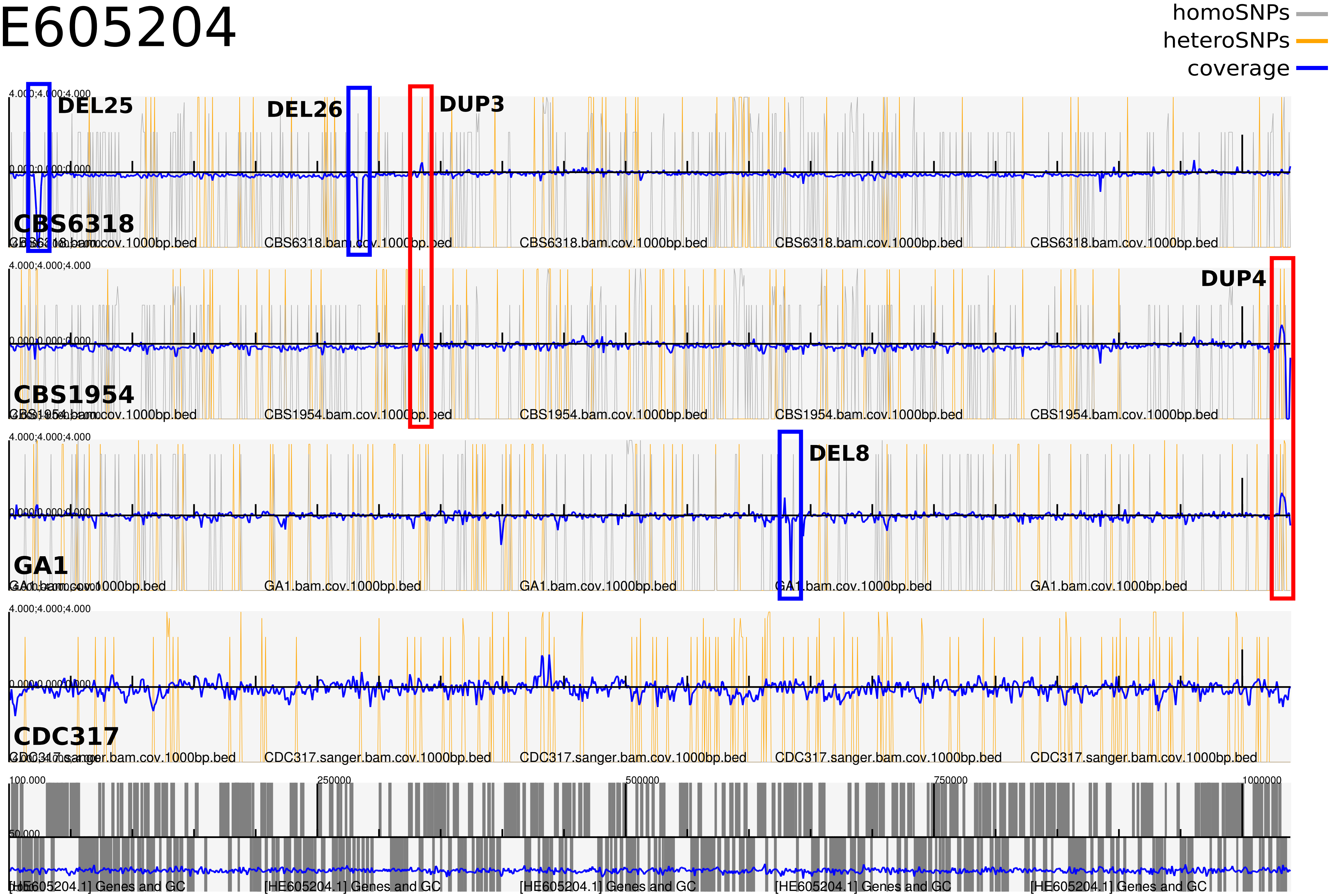
homoSNPs
heteroSNPs
coverage
DEL25
DEL26
DUP3
DUP4
DEL8
CBS6318
CBS1954
GA1
CDC317
CBS6318.bam.cov.1000bp.bed
CBS1954.bam.cov.1000bp.bed
GA1.bam.cov.1000bp.bed
CDC317.sanger.bam.cov.1000bp.bed
4.000;4.000;4.000
0.000;0.000;0.000
100.000
50.000
250000
500000
750000
1000000
[HE605204.1] Genes and GC

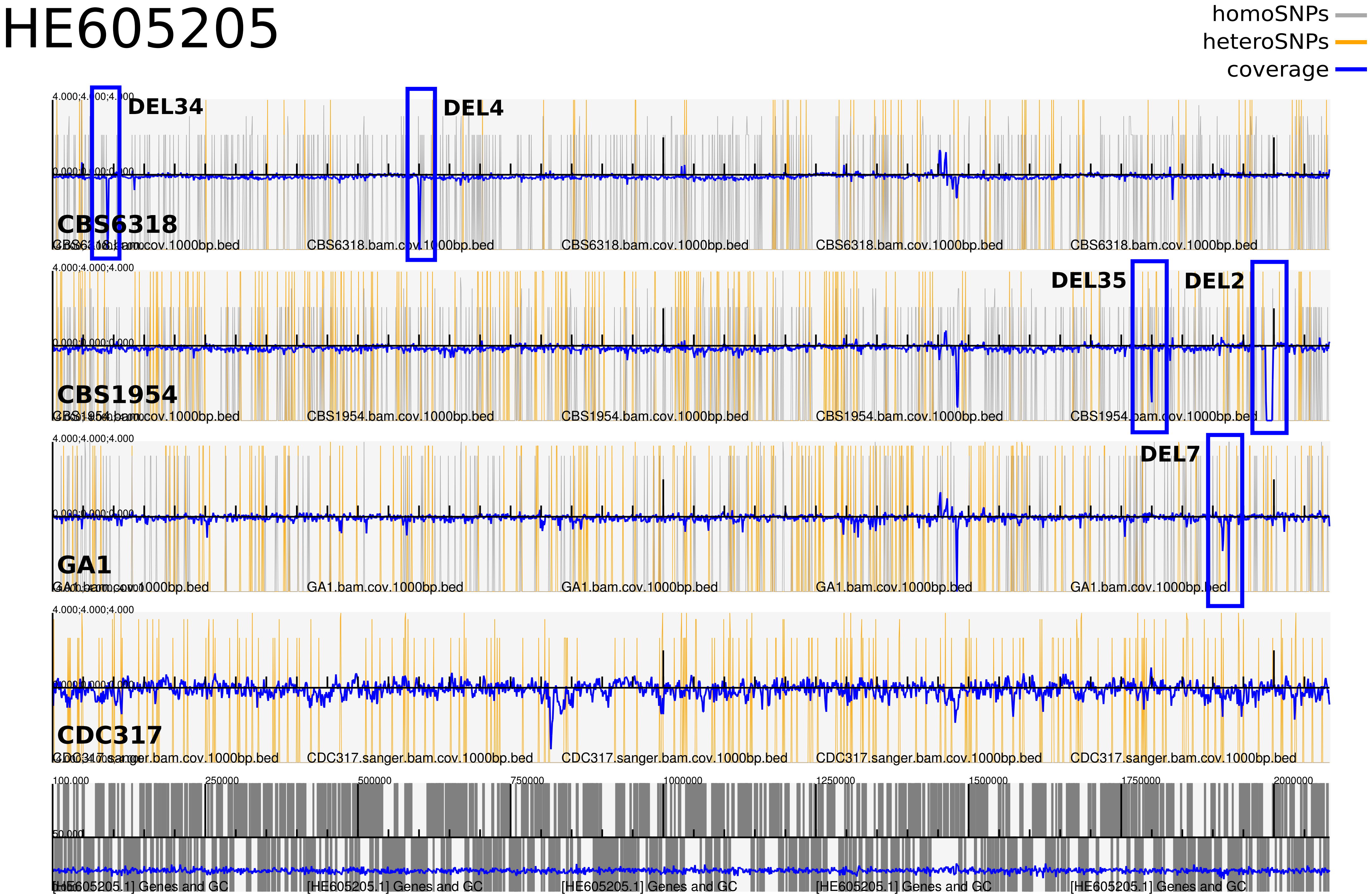
HE605205
homoSNPs
heteroSNPs
coverage
DEL34
DEL4
CBS6318
CBS6318.bam.cov.1000bp.bed
DEL35
DEL2
CBS1954
CBS1954.bam.cov.1000bp.bed
DEL7
GA1
GA1.bam.cov.1000bp.bed
CDC317
CDC317.sanger.bam.cov.1000bp.bed
250000
500000
750000
1000000
1250000
1500000
1750000
2000000
[HE605205.1] Genes and GC

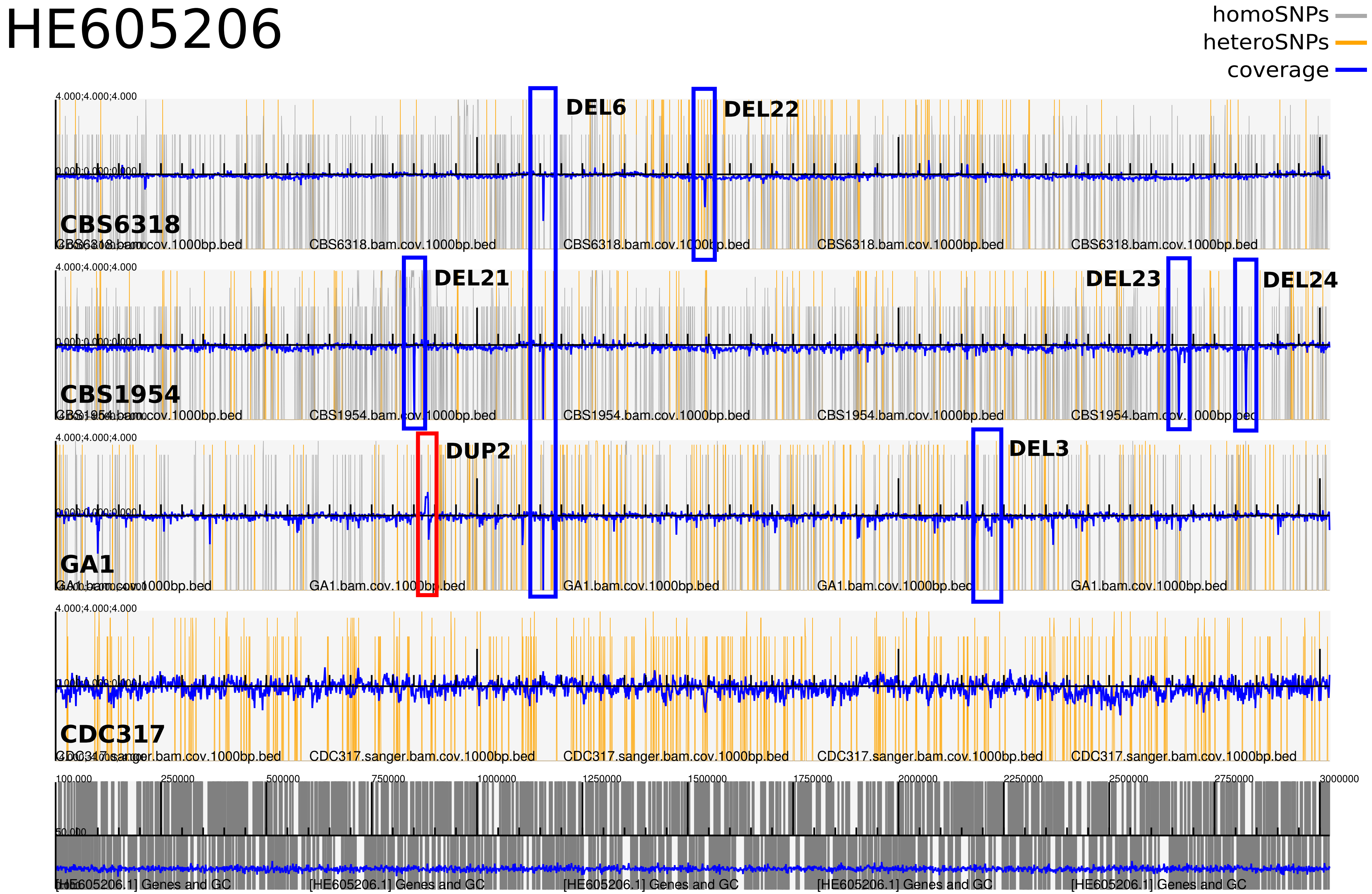
HE605206
homoSNPs
heteroSNPs
coverage
DEL6
DEL22
DEL21
DEL23
DEL24
DUP2
DEL3
CBS6318
CBS1954
GA1
CDC317
CBS6318.bam.cov.1000bp.bed
CBS1954.bam.cov.1000bp.bed
GA1.bam.cov.1000bp.bed
CDC317.sanger.bam.cov.1000bp.bed
[HE605206.1] Genes and GC
250000
500000
750000
1000000
1250000
1500000
1750000
2000000
2250000
2500000
2750000
3000000

HE605207

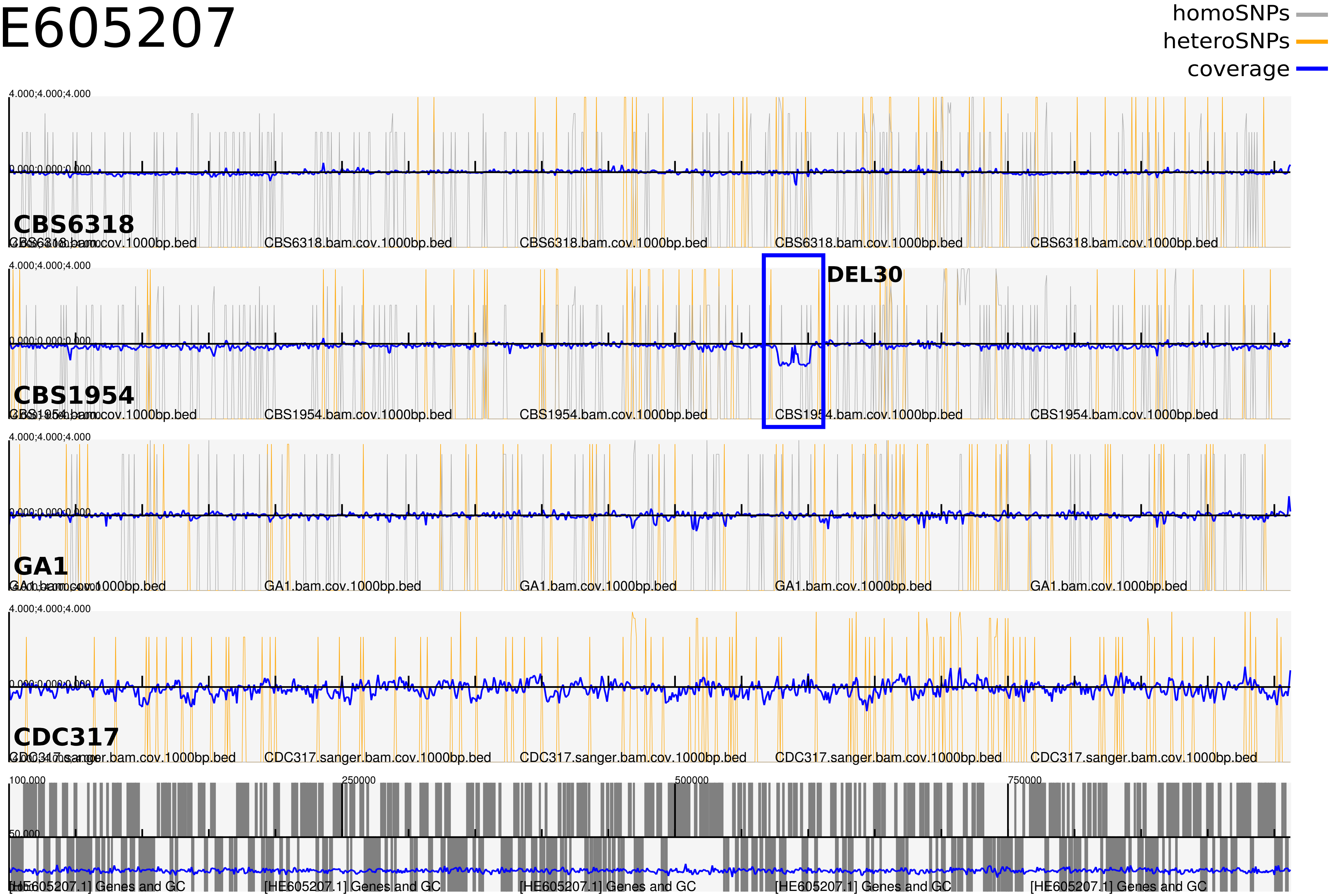
homoSNPs
heteroSNPs
coverage
4.000;4.000;4.000
0.000;0.000;0.000
CBS6318
CBS6318.bam.cov.1000bp.bed
CBS1954
CBS1954.bam.cov.1000bp.bed
DEL30
GA1
GA1.bam.cov.1000bp.bed
CDC317
CDC317.sanger.bam.cov.1000bp.bed
100.000
50.000
250000
500000
750000
[HE605207.1] Genes and GC

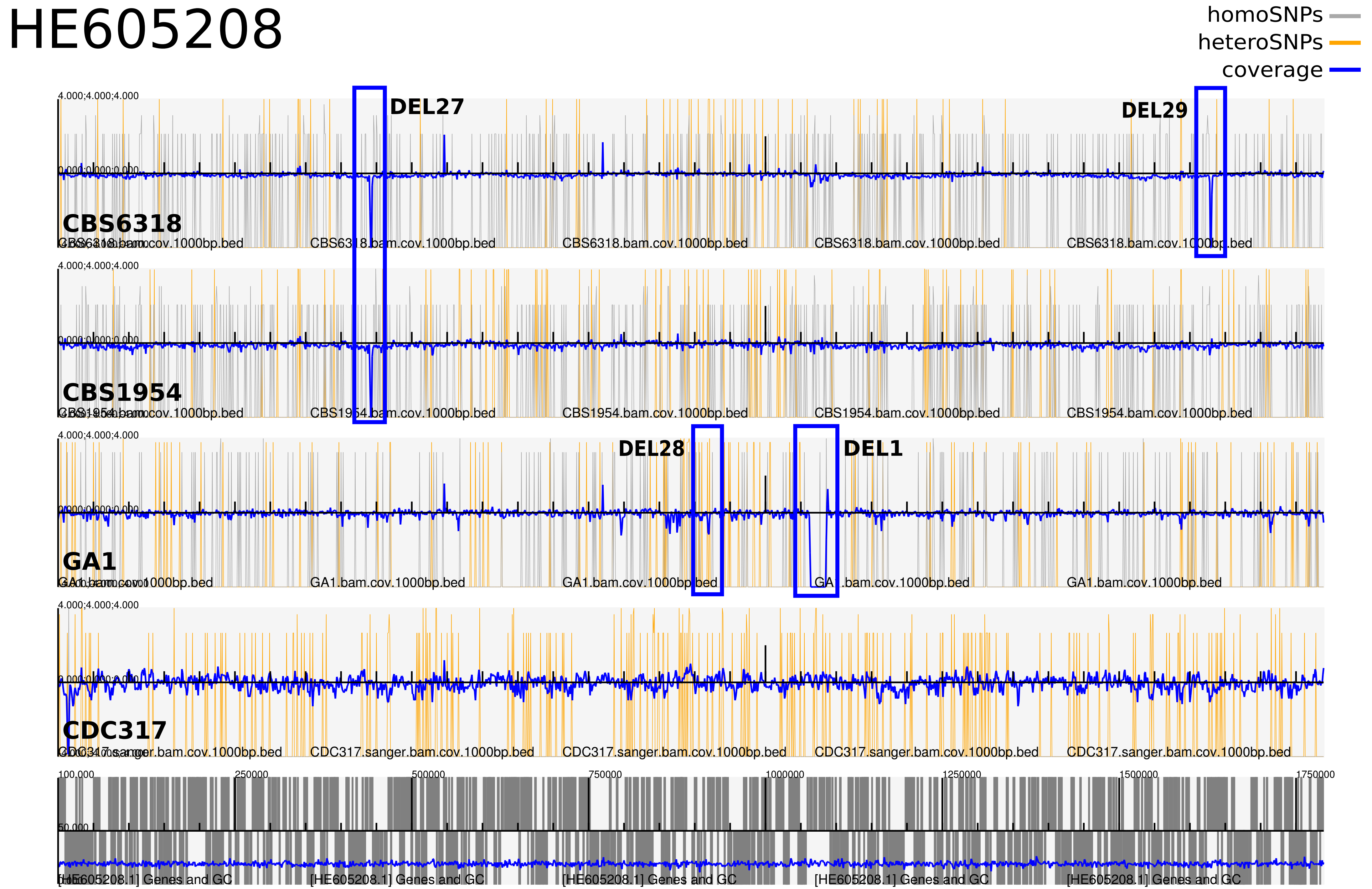
HE605208
homoSNPs
heteroSNPs
coverage
DEL27
DEL29
CBS6318
CBS1954
DEL28
DEL1
GA1
CDC317
[HE605208.1] Genes and GC

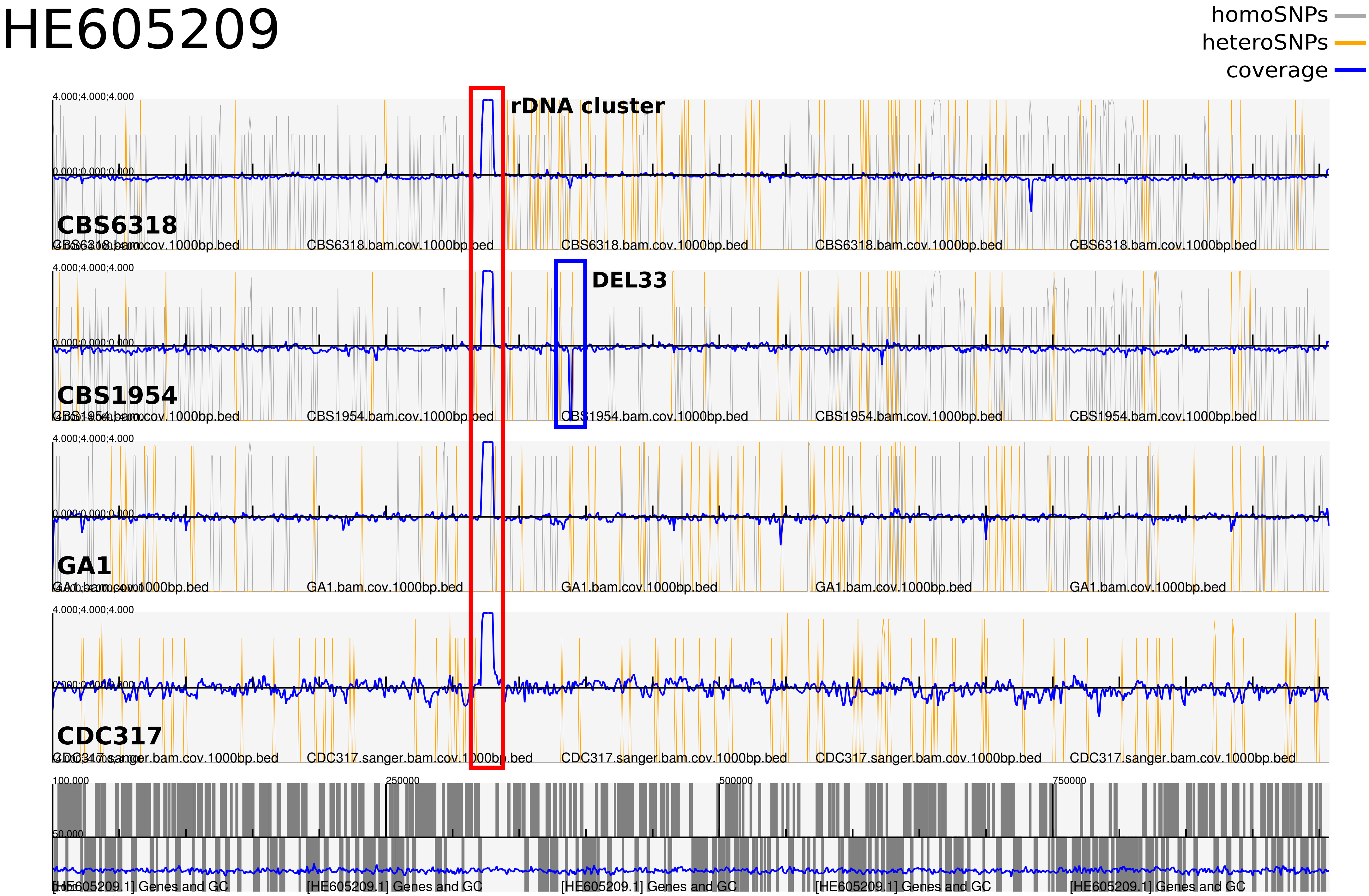
HE605209
homoSNPs
heteroSNPs
coverage
rDNA cluster
DEL33
4.000;4.000;4.000
0.000;0.000;0.000
CBS6318
CBS6318.bam.cov.1000bp.bed
CBS1954
CBS1954.bam.cov.1000bp.bed
GA1
GA1.bam.cov.1000bp.bed
CDC317
CDC317.sanger.bam.cov.1000bp.bed
100.000
50.000
250000
500000
750000
[HE605209.1] Genes and GC
