# Supplementary material for: Unexpected Genomic Variability in Clinical and Environmental Strains of the Pathogenic Yeast Candida parapsilosis
Source: Genome Biol Evol. 2013 Nov 20;5(12):2382–92. doi: 10.1093/gbe/evt185 (PMC3879973; doi:10.1093/gbe/evt185)

Supplementary file 3: Experimental validation

Experimental validation strategy.........1
PCR results for DEL#1 - DEL#20.........2
Southern Hybridization results for DEL#3, DEL#5 and DEL#6.........5
Unspecific PCR fragments for DEL#1 and DEL#6.........6

Experimental validation strategy

Detected stuctural variants have been validated experimentally by PCR and Southern hybridisation. We have designed pair of primers flanking beginning (green, PCR1) and end (blue, PCR2) of each deletion. In case there is no deletion (wt), only PCR1 (F1-R1; green) and PCR2 (F2-R2; blue) should work, while PCR3 (F1-R2) should not work or would produce much longer product (negative control, see scheme below). In contrast, if there is homozygous deletion, only PCR3 (F1-R2) should work, as F2 and R1 would have not template to anneal. In case of heterozygous deletion, all reactions (PCR1, PCR2, PCR3) should work.

Similarly, an inversion could be validated by combinations of two pairs of primers: PCR4 (F1-F2) and PCR5 (R1-R2). If there is inversion, only PCR4 and PCR5 should work. Otherwise (wt), only PCR1 (F1-R1) and PCR2 (F2-R2) should work. While in case of heterozygous inversion, both PCR1-2 and PCR4-5 should work.

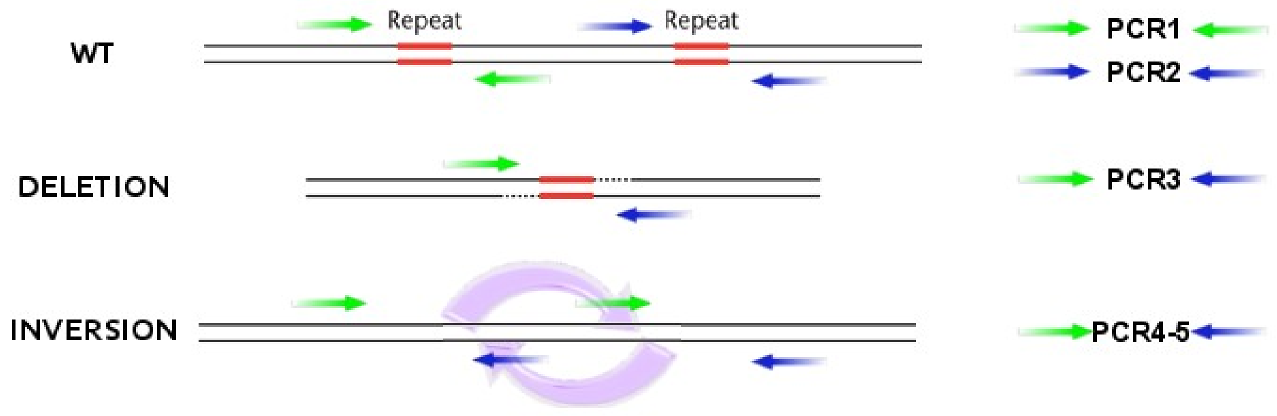

PCR results for DEL#1 - DEL#20

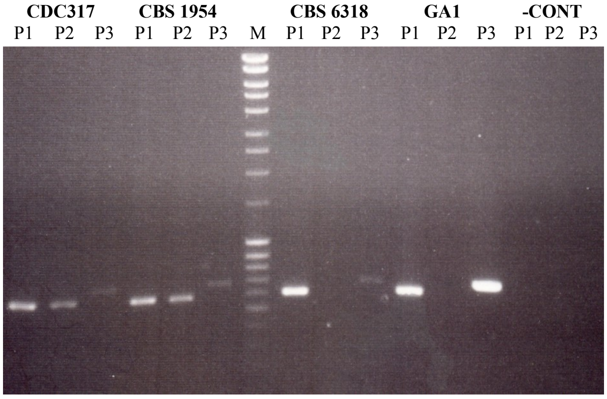

Del#1
23475 bp deletion in GA1
PCR1 - 609 bp, PCR2 - 595, PCR3 - 24161/686 bp

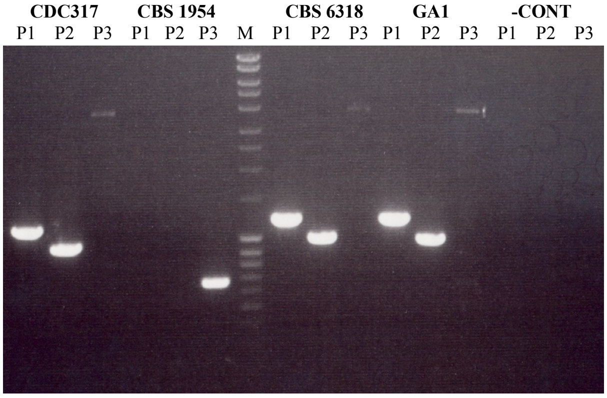

Del#2
11573 bp deletion in CBS1954
PCR1 - 1250 bp, PCR2 - 1045, PCR3 - 12247/674 bp

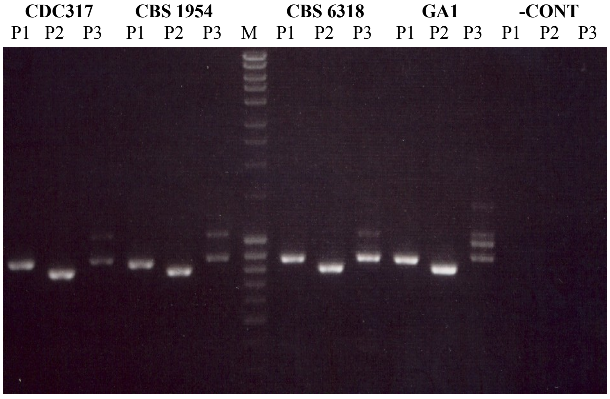

Del#3
9808 bp heterozygosity in GA1
PCR1 - 875 bp, PCR2 - 811, PCR3 - 10717/909 bp

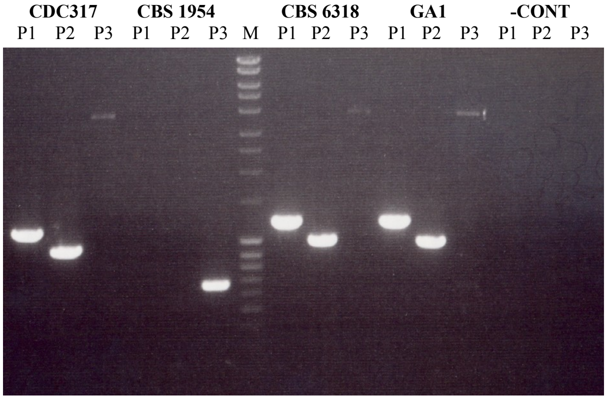

Del#4
2847 bp deletion in CBS 6318
PCR1 - 1089, PCR2 - 516, PCR3 - 3425/578 bp

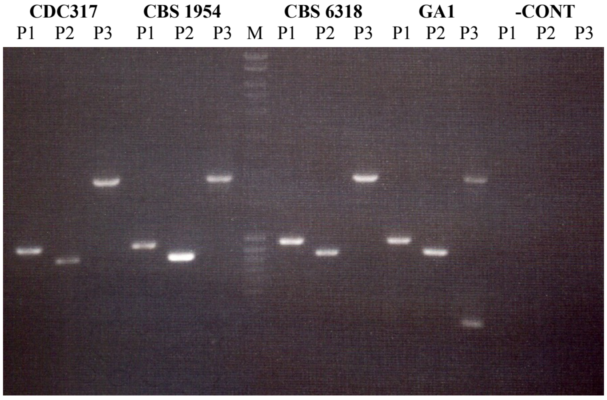

Del#5
1640 bp heterozygosity in GA1

PCR1 - 986 bp, PCR2 - 858 bp, PCR3 - 1931/291 bp

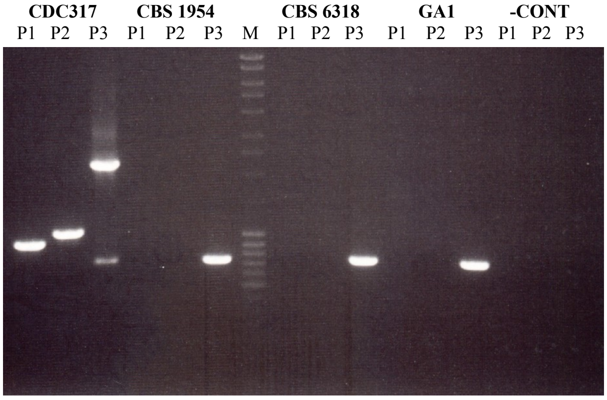

Del#6
1595 bp deletion in CBS1954, CBS6318 and GA1

PCR1 - 965 bp, PCR2 - 1090 bp, PCR3 - 2308/713 bp

CDC317 CBS 1954 CBS 6318 GA1 -CONT
P1 P2 P3 P1 P2 P3 M P1 P2 P3 P1 P2 P3 P1 P2 P3

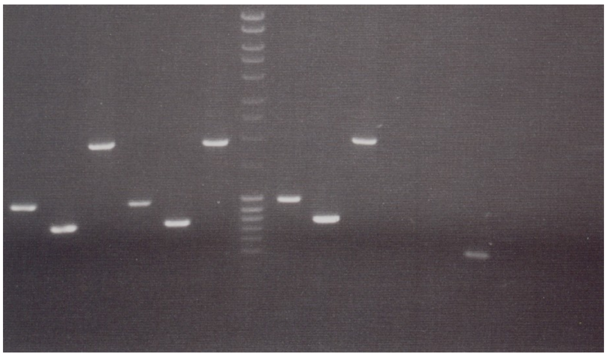

Del#7
1497 bp deletion in GA1
PCR1 - 1020 bp, PCR2 - 787 bp, PCR3 - 1932/435 bp

CDC317 CBS 1954 CBS 6318 GA1 -CONT
P1 P2 P3 P1 P2 P3 M P1 P2 P3 P1 P2 P3 P1 P2 P3

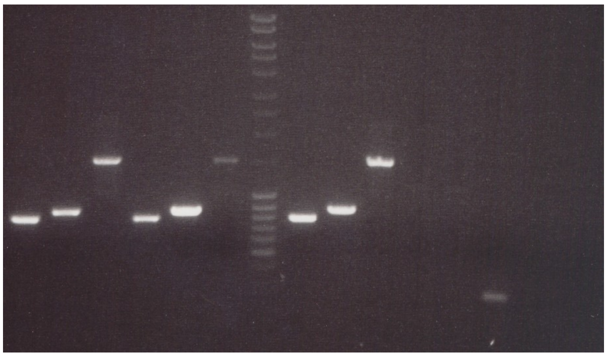

Del#8
1268 deletion in GA1
PCR1 - 818 bp, PCR2 - 902 bp, PCR3 - 1543/275 bp

CDC 317 CBS 1954 CBS 6318 GA1 - C M

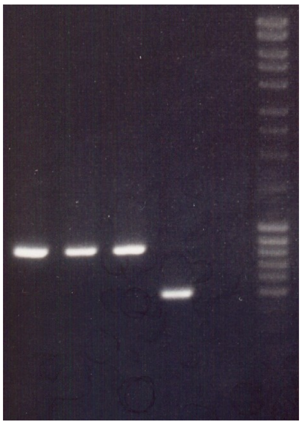

Del#9
354 bp deletion in GA1
PCR3 - 853/499 bp

CDC 317 CBS 1954 CBS 6318 GA1 - C M

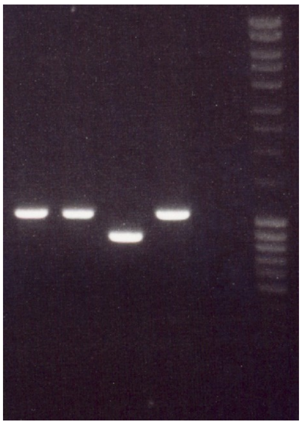

Del#10
235 bp deletion in CBS6318
PCR3 - 1104/869 bp

CDC 317 CBS 1954 CBS 6318 GA1 - C M

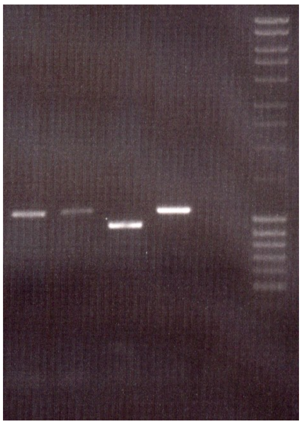

Del#11
141 bp deletion in CBS6318
PCR3 - 1118/977 bp

CDC 317 CBS 1954 CBS 6318 GA1 - C M

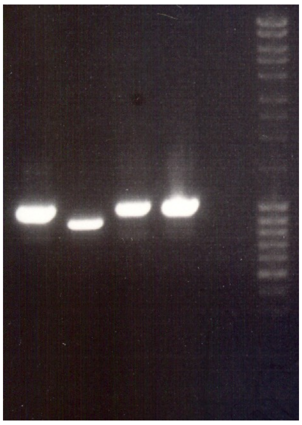

Del#12
132 bp deletion in CBS1954
PCR3 - 1092/960 bp

CDC 317 CBS 1954 CBS 6318 GA1 - C M

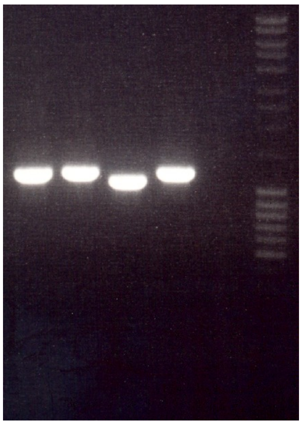

Del#13
105 bp deletion in CBS6318
PCR3 - 1220/1115 bp

CDC 317 CBS 1954 CBS 6318 GA1 - C M

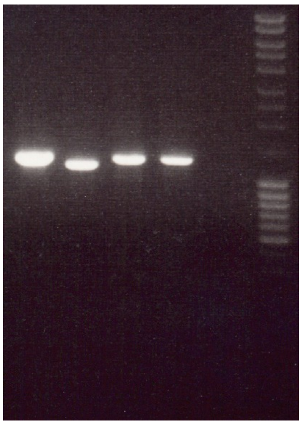

Del#14
95 bp deletion in CBS1954
PCR3 - 1298/1203 bp

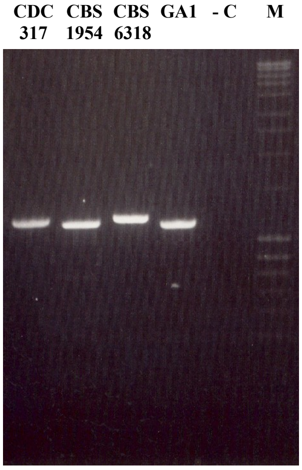

Del#15
59 bp deletion in CBS1954 and GA1
PCR3 - 1258/1199 bp

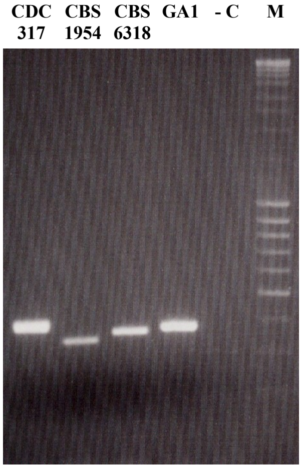

Del#16
43 bp deletion in CBS1954
PCR3 - 394/351 bp

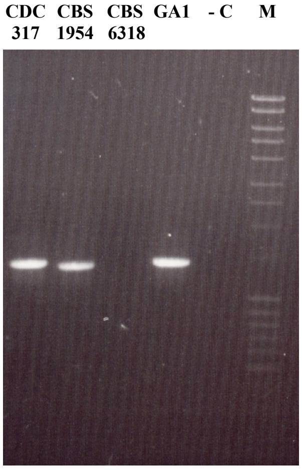

Del#17
39 bp deletion in CBS1954
PCR3 - 1430/1391 bp

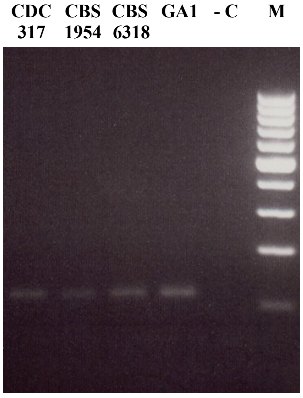

Del#18
34 bp heterozygosity in CDC317
PCR3 - 149/115 bp

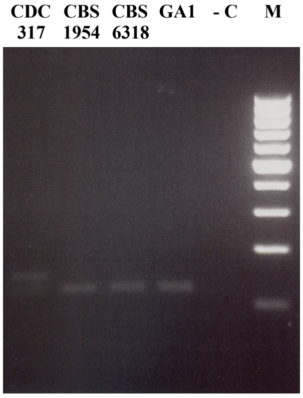

Del#19
18 bp heterozygosity in CDC317
PCR3 - 120/122 bp

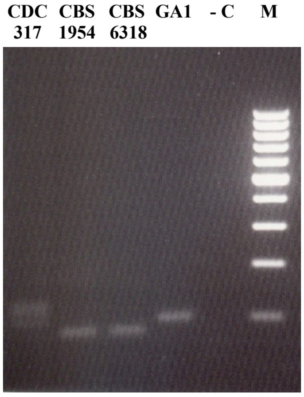

Del#20
17 bp heterozygosity in CDC317
PCR3 - 118/101 bp

M (Del#1 - Del#17): Fermentas MassRuler™ DNA Ladder Mix, ready-to-use, #SM0403: 10000, 8000, 6000, 5000, 4000, 3000, 2500, 2000, 1500, 1031, 900, 800, 700, 600, 500, 400, 300, 200, 100, 80 bp

M (Del#18 - Del#20): Fermentas GeneRuler™ 100bp DNA Ladder Plus #SM0321: 3000, 2000, 1500, 1200, 1031, 900, 800, 700, 600, 500, 400, 300, 200, 100 bp

Southern Hybridization results for DEL#3, DEL#5 and DEL#6

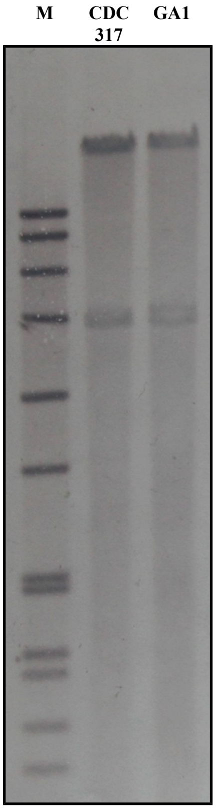

Del#3
9808 bp heterozygosity in GA1
WT: 13228 bp
HZY: 13228 and 3514 bp

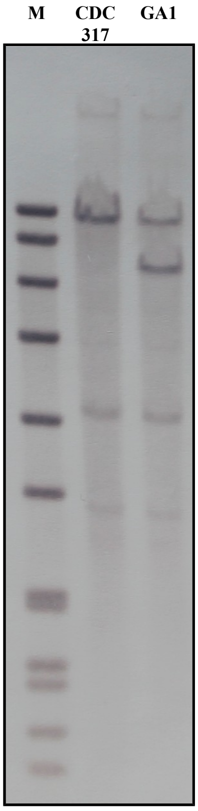

Del#5
1640 bp heterozygosity in GA1
WT: 8161 bp
HZY 8161 and 6513 bp

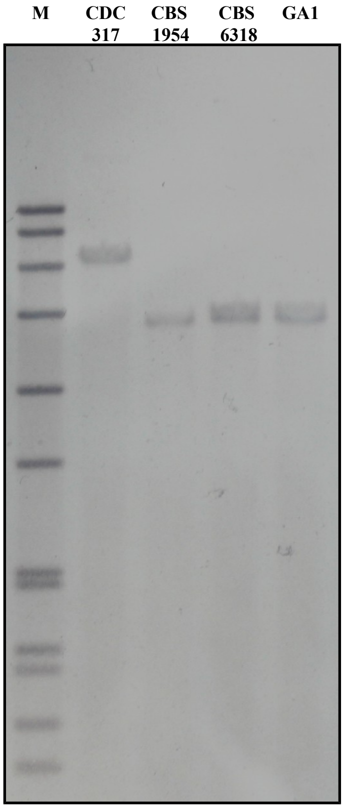

Del#6
1595 bp deletion in
CBS1954, CBS6318 and GA1
WT: 6167 bp
Δ: 4617 bp

M: Roche DIG-labeled DNA Molecular Weight Marker VII 0.081 - 8.57 kbp: 8576, 7427, 6106, 4899, 3639, 2799, 1953, 1882, 1515, 1482, 1164, 992, 718, 710, 492, 359, 81 bp

Unspecific PCR fragments for DEL#1 and DEL#6

Although Deletion #1 was identified in GA1 *in silico*, weak PCR3 products for CDC317, CBS1954 and CBS6318 were also detectable. These seemed to have the same size as the one from GA1. The BLAST analysis revealed unique sites of primer binding, thus the unexpected fragments were not the consequences of unspecific primer binding. The detailed analysis of this region revealed a 188 nt long duplicated sequence (differed only in 3 nucleotides) up- and downstream from the breakpoints of the deletion. The breakpoints divided this sequence into a 79nt long upstream part and a 109 nt long downstream part (see the scheme below, 1). *In silico* analysis established that the primers bind to the DNA outside of these. According to these observations, we hypothesised that during the chain reaction incomplete products, containing above mentioned 188 nt region, are synthesized from both primers (2). In the following PCR cycle, these newly produced fragments can bind randomly to any of two regions around the breakpoints (3). In fifty percent of the cases by the end of this cycle the elongation gets finished causing that the emergent product also has the binding site of the opposite primer (4). During the next cycles this fragment can serve as a template leading to the concentration of a false positive PCR3 fragment in wild-type strains (5).

Deletion #6 was found in CBS1954, CBS6318 and GA1 *in silico* and was verified by PCR and Southern-blot strategy. PCR3 reaction of CDC317 wild-type resulted in a large fragment corresponding to the expected size (2308 bp). However, smaller unexpected product also appeared. The evidences suggested the same phenomenon as we experienced in the case of Deletion #1. Examination of this region identified similar genomic background: 288 nt long similar sequence (differed only in 4 nucleotides) was identified in the region of the breakpoints that divided it into a 259 nt upstream and a 29 nt downstream part. The false positive fragment could be synthesized in the same way as we described above.

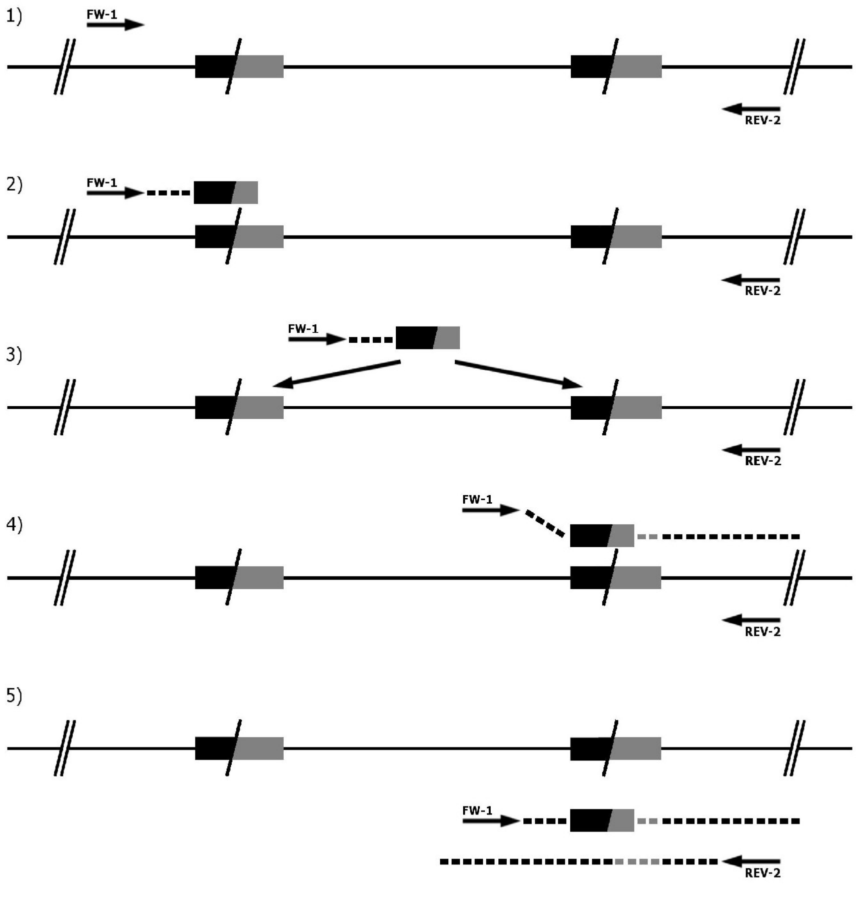

Supplement: Supplementary Data [file supp_evt185_SuppFile4.pdf]
